# Genome-Wide Gene Expressions Respond Differently to A-subgenome Origins in *Brassica napus* Synthetic Hybrids and Natural Allotetraploid

**DOI:** 10.3389/fpls.2016.01508

**Published:** 2016-10-13

**Authors:** Dawei Zhang, Qi Pan, Chen Tan, Bin Zhu, Xianhong Ge, Yujiao Shao, Zaiyun Li

**Affiliations:** ^1^National Key Lab of Crop Genetic Improvement, National Center of Crop Molecular Breeding Technology, National Center of Oil Crop Improvement, College of Plant Science and Technology, Huazhong Agricultural UniversityWuhan, China; ^2^Key Laboratory of Ecological Remediation and Safe Utilization of Heavy Metal-Polluted Soils, College of Life Science, Hunan University of Science and TechnologyXiangtan, China; ^3^College of Chemistry and Life Science, Hubei University of EducationWuhan, China

**Keywords:** *Brassica napus*, RNA-Seq, additive gene expression, expression level dominance, allopolyploidization

## Abstract

The young allotetraploid *Brassica napus* (2*n* = 38, AACC) is one of models to study genomic responses to allopolyploidization. The extraction of AA component from natural *B. napus* and then restitution of progenitor *B. rapa* should provide a unique opportunity to reveal the genome interplay for gene expressions during the evolution. Herein, *B. napus* hybrids (2*n* = 19, AC) between the extracted and extant *B. rapa* (2*n* = 20, AA) and the same *B. oleracea* genotype (2*n* = 18, CC) were studied by RNA-seq and compared with natural *B. napus* donor, to reveal the gene expression changes from hybridization and domestication and the effects of A genome with different origins. Upon the initial merger of two diploid genomes, additive gene expression was prevalent in these two hybrids, for non-additively expressed genes only represented a small portion of total expressed genes. A high proportion of genes exhibited expression level dominance, with no preference to either of the parental genomes. Comparison of homoeolog expressions also showed no bias toward any genomes and the parental expression patterns were often maintained in the hybrids and natural allotetraploids. Although, the overall patterns of gene expression were highly conserved between two hybrids, the extracted *B. rapa* responded less and appeared more compatible for hybridization than the extant *B. rapa*. Our results suggested that expression level dominance and homoeolog expressions bias were balanced at the initial stage of genome merger, and such balance were largely maintained during the domestication of *B. napus*, despite the increased extent over time.

## Introduction

Allopolyploidization, through the merger and duplication of two or more sets of divergent parental genomes, is an ancient and ongoing evolutionary process (Otto, 2007; Doyle et al., 2008). The prevalence of allopolyploids in flowering plants, including many important crops, such as oilseed rape (*Brassica napus*), cotton (*Gossypium* ssp.), wheat (*Triticum aestivum*), suggests the evolutionary advantage of allopolyploids in ecological adaptation, and agricultural production over their progenitors (Chen, 2007, 2010). Indeed, an array of investigations in recent or synthetic allopolyploids have demonstrated that the initial stage of allopolyploidization is accompanied by various changes at genetic (Song et al., 1995; Xiong et al., 2011), epigenetic (Adams et al., 2003; Cui et al., 2013; Ge et al., 2013) as well as gene expression level (Wang et al., 2006; Chelaifa et al., 2010; Yoo et al., 2013). Such profound changes may enhance the fitness and adaptability, thus enable allopolyploids to survive in novel environments not accessible to their parent species (Li et al., 2014).

Genome-wide expression changes have been widely demonstrated in natural and synthetic allopolyploids using microarrays and RNA-seq. These methods provide the most comprehensive data that we have so far and facilitate our understanding of gene expression patterns in allopolyploids. An initially and well explored issue is whether the gene expression levels observed in allopolyploid are equal to the value average from that in its progenitors (additive), or not (non-additive). Although, additive expression is prevalent, many of these expression changes are non-additive in allopolyploids where expression levels deviate from the MPV (mid-parent expression values, Wang et al., 2006; Chagué et al., 2010; Yoo et al., 2013; Zhao et al., 2013). Notably, two recent studies in synthetic wheat allohexaploids that combine the AB genome extracted from natural hexaploid wheat suggest that a majority of genes were additively expressed and the extracted tetraploid component appears more compatible for hybridization than natural tetraploid (Chelaifa et al., 2010; Zhang et al., 2014). In addition to non-additive expression, homoeolog expression bias, where the two homoeologs are expressed unequally, is commonly observed in allopolyploids, but varies among tissues and species (Chelaifa et al., 2010; Flagel and Wendel, 2010; Yoo et al., 2013; Chalhoub et al., 2014; Li et al., 2014). Moreover, homoeolog expression bias observed in parents could be maintained in the allopolyploid derivatives, indicating that these expression changes are heritable (Flagel and Wendel, 2010; Yoo et al., 2013; Li et al., 2014). Strikingly, recent finds have shown that the expression levels of a large proportion of genes in allopolyploid might be statistically similar to one parent but differential from the other parent (Chagué et al., 2010; Chelaifa et al., 2010; Bardil et al., 2011; Li et al., 2014). This intriguing phenomenon which was first described in cotton is now referred as expression level dominance (Rapp et al., 2009). As with homoeolog expression bias, expression level dominance may be contingent upon tissue type and environmental conditions, whereas the mechanism remains unclear (Bardil et al., 2011; Yoo et al., 2013). Importantly, both homoeolog expression bias and expression level dominance are quantitative, meaning that they may be balanced, or unbalanced, alternatively (Grover et al., 2012).

*Brassica napus* (2*n* = 4x = 38, AACC) is an important oilseed crop and widely grown over the world, which was formed by recent allopolyploidy (~7500 years ago) between *B. rapa* (2*n* = 2x = 20, AA) and *B. oleracea* (2*n* = 2x = 18, CC). Its two subgenomes showed subtle structural changes and incipient gene loss, including abundant homeologous exchanges (Chalhoub et al., 2014). One restituted *B. rapa* genotype from natural *B. napus* by extracting A-subgenome (Tu et al., 2010) showed certain genetic divergence from extant *B. rapa* accessions (Guo et al., 2014), which provides the unique opportunity to address the interesting question how the evolved A genome responds again to the genome merger with C genome and behaves in the ways similar to or different from the A genome of extant *B. rapa*. Resynthesized *B. napus* through the interspecific hybridizations between the extant diploids closely related to two progenitors have been widely investigated by different methods (cDNA-AFLP, microarrays as well as RNA-seq), which provided many new insights into the genetic and gene expression changes during the initial stage of allopolyploid formation (Xiong et al., 2011; Cui et al., 2013; Jiang et al., 2015; Zhang D. et al., 2015). However, previous studies on gene expression changes mainly focus on differential and non-additive expression, but expression level dominance and homoeolog expression bias documented for other allopolyploids are barely explored in *B. napus*.

**Figure 1:**
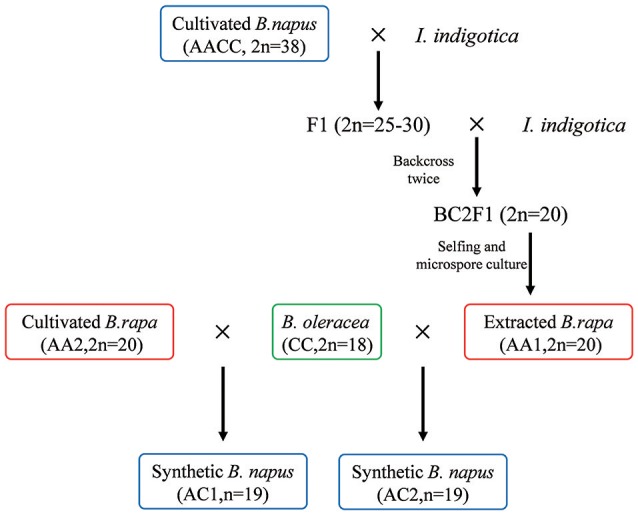
**Schematic presentation of the plant materials used in the study**. The extracted *B. rapa* (AA1) is derived from natural *B. napus* (AACC, 2*n* = 38, cultivar “Oro”) after pollination by *I. indigotica* twice and selfing for generations. The two synthetic hybrids are obtained through hybridization between the extracted and cultivated *B. rapa* and the same *B. oleracea* without chromosome doubling. Plant materials used in the study are indicated by differential colored circle. The same colors are also used in the subsequent figures.

In present study, gene expression analyses of two synthetic *B. napus* hybrids (between the extracted and natural *B. rapa* and the same *B. oleracea*) and the donor *B. napus* for extraction, are performed to better understand the effects of hybridization and domestication process on global gene expression architecture (Figure 1). Besides differential and non-additive expression described in previous studies, we also assess what extent and direction of expression level dominance and homoeolog expression bias, whether these expression changes are consistent between the two type *B. napus* hybrids, and how hybridization and domestication have contributed to these expression changes.

## Materials and methods

### Plant materials

To analyze the immediate gene expression changes after the genome merger in synthetic *B. napus*, two F_1_ hybrids (AC, *n* = 19) between *B. rapa* (as female parent) and *B. oleracea* (as male parent) without chromosome doubling were produced. The first one (referred as AC1) was synthesized from the cross between the DH line of the restituted *B. rapa* (AA1, 2*n* = 20) and *B. oleracea var. alboglabra* (CC, 2*n* = 18, genotype Chi Jie Lan) inbred lines. The new *B. rapa* DH lines were derived from backcross twice between natural *B. napus* (AACC, 2*n* = 38, cultivar “Oro”) and Chinese woad (*Isatis indigotica*, 2*n* = 14). *Isatis indigotica*, which belongs to the tribe Isatideae of the family Brassicaceae, has been used as a medicinal plant since ancient times in China. After *B. napus* cv. Oro was pollinated by *I. indigotica*, the non-classical hybrids obtained had the variable chromosome numbers (2*n* = 19–38) from *B. napus* and few alien chromosomes or DNA sequences. The chromosomes from the pollinator Chinese woad were likely eliminated mostly during certain stages of embryo or hybrid plant development, because of their distant relationships with *B. napus*. Relevantly for the extraction of A-subgenome, some hybrids (2*n* = 29) lost some C-subgenome chromosomes but kept all the chromosomes of the A genome. After two more rounds of the pollinations by Chinese woad to induce chromosome eliminations in progenies, A-subgenome was extracted and the ancestral *B. rapa* was restituted (Figure 1; Tu et al., 2010). The completeness of A-subgenome in the restituted *B. rapa* was shown by cytological and genetic studies (Figure 1; Tu et al., 2010; data not shown). The second F_1_ hybrid (AC2) was previously generated from the cross of another *B. rapa* (genotype 3H120) inbred line and the same *B. oleracea* genotype above (Cui et al., 2012). These two hybrids were derived from single immature embryo culture on MS medium without hormone. All the plants were grown under the same conditions to minimize the developmental or environmental effects.

### RNA extraction and library preparation

The newly emerged and expanded young leaves (third-leaf stage) from two plants were collected, frozen in liquid nitrogen and stored at −80 until RNA extraction. RNA was extracted from two biological replicates using TRIzol reagent (Invitrogen, Life Technologies) following standard protocol. The quality and quantity of the extracted RNA were checked for integrity on the Agilent Technologies 2100 Bioanalyzer (Agilent) according to their RNA Integrity Number (RIN) value. RNA-Seq library construction was processed following TruSeq RNA Sample Prep v2 protocol. The libraries were subsequently sequenced on Illumina HiSeq™ 2000 with 100 bp paired-end reads at Huazhong Agricultural University (Wuhan, China).

### Reads filtering and mapping

After the high-throughput sequencing, the raw data (AA1, 22048313; AA2, 21937437; CC, 24351000; AC1, 37419334; AC2, 27341175) which contained adapters were trimmed and low quality reads were filtered. Then the clean and properly paired reads (AA1, 14031130; AA2, 15043543; CC, 16462145; AC1, 25381592; AC2, 18454666) were then aligned to the *B. napus* reference genome (Chalhoub et al., 2014) using Burrows-Wheeler Alignment (BWA version: 0.7.5a-r405) with the default parameters. Mapped reads were then filtered using SAMtools (version:0.1.12a) and only the best unique mapped reads were used for further study to provide sensitive and accurate results. RNA-Seq reads were count-filtered using the standards which were adjusted from Chalhoub et al. (2014), as follows: (1) mapping best unique BWA match; (2) mapping read1 and read2 on the same gene with coherent orientations; (3) mapping of read1 and read2 on adjacent genes. Other cases such as one single end mapped on a gene or both ends mapped on non-adjacent gens were not considered. The gene expression level was calculated for all succeeding analyses using FPKM method to reduce the effect of different length between homeologous gene pairs. Among the 101040 genes in the *B. napus* assembly, an average 46.4% (AA1, 40068; AA2, 41908; CC, 40232; AC1, 54338; AC2, 53436; AACC, 51457) were expressed (FPKM > 0) in leaf.

### Analysis of differentially expressed genes

Differential expression analysis was performed between pairwise samples using DEGseq by the FET (Fisher' exact tests) method (Wang et al., 2010). The raw *P*-values were adjusted for multiple testing by the BH method (Benjamini and Hochberg, 1995) and genes were declared differentially expressed between two samples if the adjusted *P*-values were less than 0.05.

To assess how the extent and direction of expression level dominance, we compared expression levels across natural allopolyploid/synthetic hybrids and their diploid progenitors, as well as the *in silico* mid-parent expression values (MPV, average of expression levels of their parents). Cross comparisons with classification based on differential expression (*P* < 0.05), or not (*P* ≥ 0.05), led to 9 major expression categories (AC vs. MPV, AA vs. CC; **Figure 3B**) and 19 possible expression patterns (AA vs. CC, AA vs. AC, AC vs. CC; **Figure 3A**) according to Chagué et al. (2010).

For analysis of homoeolog expression bias, we selected the homoeologous gene pairs between A and C sub-genomes as described in the available reference genome sequence data to determine for which the direction of the homoeolog was biased (Chalhoub et al., 2014). In addition, comparisons between their progenitors were made to estimate whether the differentially expressed gene pairs in hybrids were a legacy of expression differences that were already present in the diploid species.

Considering the high homology between the genes in *A. thaliana* and *B. napus*, the orthologous genes in *A. thaliana* were used to predict the most probable function of the gene pairs. GO enrichment analysis was performed using the web-based AmiGO2 software (http://amigo.geneontology.org/amigo). GO terms or slims with corrected *P* < 0.05 were considered to be significantly enriched.

### Validation of the expression data by qRT-PCR

The RNA samples used for the qRT-PCR assays were the same as for the RNA-seq experiments. First-strand cDNA synthesis was performed with 1500 ng of total RNA using Thermo Scientific RevertAid First Strand cDNA Synthesis Kit, total RNA (0.5 μg) was reverse-transcribed with oligo (dT)18 primer (0.5 μg/μl) according to the described protocol. Gene-specific primers were designed according to the reference unigene sequences using the Primer 3.0, all primer sequences are listed in Table S6. A primer was also designed for *B. napus* actin gene to normalize the amplification efficiency. qRT-PCR assays in triplicate were performed using Kapa Probe Fast qPCR Kit with a Bio-Rad CFX96 Real-Time Detection System. The actin gene was used as an internal control for data normalization, and quantitative variation in the different replicates was calculated using the delta-delta threshold cycle relative quantification method.

## Results

### Differential and non-additive gene expressions in *B. napus* hybrids

**Figure 2:**
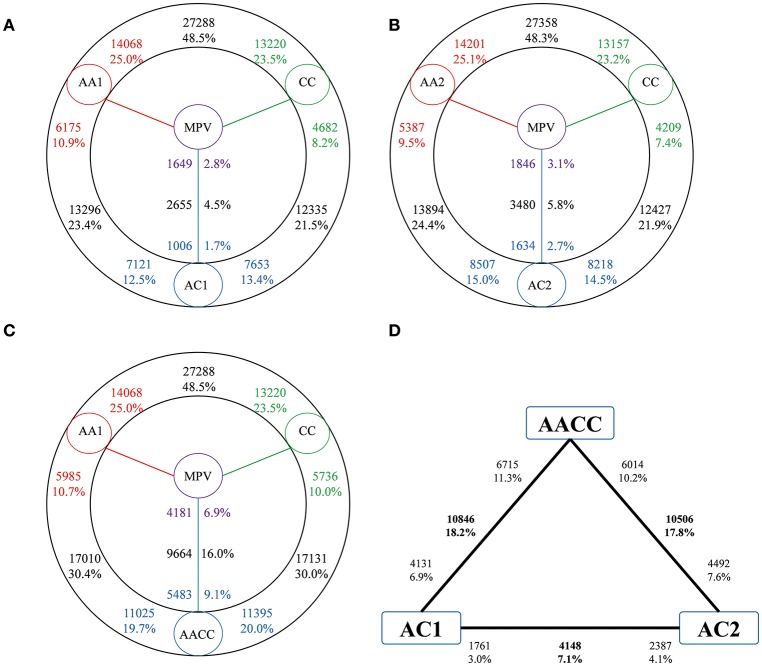
**Differential gene expressions among hybrids and their progenitors**. The total number of genes (black) differentially expressed is given in each contrast. Number close to the species (colored) represent up-regulated compared with the adjacent species. These percentages are calculated by dividing the total number of expressed genes in two adjacent species. Red, A genome progenitor; green, C genome progenitor; blue, hybrids; purple, MPV. **(A)** Hybrid AC1. **(B)** Hybrid AC2. **(C)** Natural *B. napus* AACC. **(D)** Cross comparisons between two hybrids and natural *B. napus*.

An average of 26,619,451 raw reads were obtained and 17,874,615(67.0%) properly paired reads were mapped to reference genome. To study the gene expression patterns after the genome merger, we first performed pairwise comparisons between the two progenitors involved in each cross to identify pre-existing divergence in gene expression (Figure 2). As a result, the gene expression showed remarkable divergence as nearly one half of the expressed gene were differentially expressed between A and C genome progenitors (48.5% between AA1 and CC, 48.3% between AA2 and CC, respectively, Figures 2A,B). However, the percentage of genes exhibiting high expression between two parental diploids was symmetric (*P* > 0.05, Fisher's exact test).

Comparisons between synthetic hybrids/natural *B. napus* and parental diploids also showed a high fraction of differential expressed genes, with equivalent proportions (23.4 vs. 21.5% in AC1; 24.4 vs. 21.9% in AC2 and 30.4 vs. 30.0% in AACC, respectively) and no significant bias toward either parental genome (*P* > 0.05, *t*-test). Whereas there was a higher proportion of up than down-regulated genes in hybrids and natural allopolyploid relative to parental diploids (*P* < 0.05, *t*-test).

We then compared gene expression level between synthetic hybrids/natural allopolyploid and the *in silico* mid-parent expression values (MPV) to assess non-additive expression (Figures 2A–C). The majority of genes were of additivity (95.5% in AC1 and 94.2% in AC2, respectively), and non-additively expressed genes only represented a small portion of expressed genes (4.5% in AC1 and 5.8% in AC2) in synthetic hybrids. The overall distribution of most non-additively expressed genes along the chromosomes seemed to be completely random. However, in some chromosomes (A6 and C5 in AC1), large regions of down-regulated genes were observed, suggesting that non-additive expression was not randomly organized but followed specific patterns along some chromosomes (Figure S1).

Despite the same A genome and cytoplasm shared, the number of differentially expressed genes between AC1 and AACC was greater than that between the two hybrids, suggesting that genome doubling and domestication had great impact on gene expression (10846 vs. 4148; Figure 2D).

### Comparison of gene expressions between synthetic hybrids

Cross comparisons between synthetic hybrids (AC1 vs. AC2), as well as their A genome progenitors (AA1 vs. AA2) were made to address whether the differential expression observed in synthetic hybrids simply reflect the vertical transmission of preexisting expression differences (Figure S2). Among 43847 shared genes, approximate 86.0% (37696) were equally expressed in both synthetic hybrids and the progenitors. Meanwhile, 453 and 461 of these differential expressed genes were commonly up and down-regulated, respectively, in both comparisons. These shared genes were further functionally classified into Gene Ontology (GO) slims (Table S1; *P* < 0.05). Genes involved in catalytic activity, metabolic process were up-regulated, whereas those involved in structural molecule activity, oxidoreductase activity and protein metabolic process were down-regulated in both AA1 and AC1 as compared with AA2 and AC2.

We also compared sets of additively and non-additively expressed genes revealed in AC1 and AC2. The majority of genes exhibited similar expression patterns between hybrids (Figure S3). This included 51863 (91.6%) genes that were additively expressed, and 825 (1.5%; 254 up and 571 down-regulated) genes that were non-additively expressed in both hybrids as compared with MPV. However, 2167 (3.8%; 1114 up and 1053 down-regulated) genes showing additive expression in the AC1 displayed non-additive expression in AC2; Inversely, 1388 (2.5%; 523 up and 865 down-regulated) genes showed expression changes from non-additive to additive. Only 183 (0.3%) and 186 (0.3%) non-additively expressed genes which were up and down-regulated in the AC1 showed opposite expression patterns in AC2, respectively. In addition, GO analysis was performed for those non-additively expressed genes in the two hybrids and the details were provided in Table S2.

### Classification of gene expression patterns

**Figure 3:**
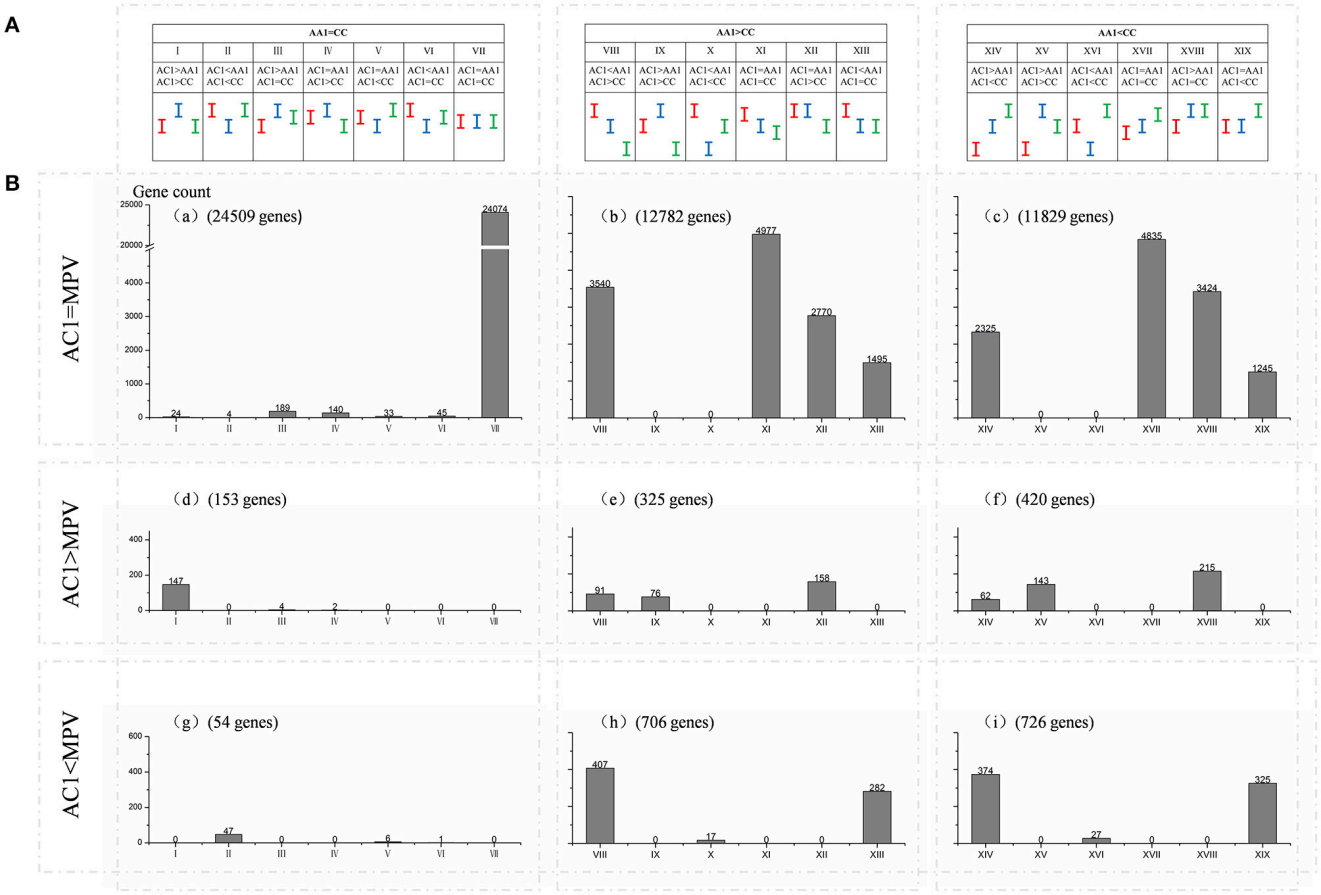
**Global classification of gene expression among hybrid AC1 and its progenitors**. Genes based on differentially or non-differentially among the hybrid, diploid progenitors, and MPV, were further classified into **(A)** 19 possible expression patterns (I—XIX; AA1 vs. CC, AA1 vs. AC1, AC1 vs. CC) and **(B)** 9 major expression categories (a–i; AC1 vs. MPV, AA1 vs. CC). Red, green and blue bars represent the confidence intervals of expression level observed in A and C genome progenitors and hybrid, respectively. Genes are differentially expressed between two samples when their confidence intervals (bars) do not overlap whereas it is equal when they do overlap. The x-axis represents the 19 possible expression patterns. The y-axis represents gene count. The number of genes for each expression pattern in each of expression categories is also indicated.

As statistical comparisons were made among the allopolyploid/hybrids, diploid progenitors, and MPVs, we further classified genes into 19 possible expression patterns (I—XIX; AA vs. CC, AA vs. AC, AC vs. CC; Figure 3A) and 9 major expression categories (a–i; AC vs. MPV, AA vs. CC; Figure 3B) following the method of Chagué et al. (2010). In general, there were more down-regulated genes (1486, categories g, h, i) than up-regulated (898, categories d, e, f) in hybrid AC1, in comparison with MPV (*P* < 0.05, *t*-test). Expression levels of the majority genes in the hybrid were equal to those of both progenitors (33886, patterns VII, XI, XVII) or those of one of them (10334, patterns III, IV, V, VI, XII, XIII, XVIII, XIX). Besides that, a fraction of the total genes exhibited transgressive expression, for which the expression levels were statistically elevated or depressed relative to the two parents (485; patterns I, II, IX, V, XV, XVI). Among them, more genes displayed transgressive up- (390, patterns I+IX+XV) than down-regulation (95, patterns II+X+XVI) in hybrid (*P* < 0.05, *t*-test). Similar expression patterns were observed in AC2, indicating a high conservation of gene expression between the two hybrids (Figure S4). However, more transgressive expression was also found in natural allopolyploid relative to synthetic hybrids, especially transgressive up-regulation (3144 up vs. 298 down regulation, Table S3).

### Expression level dominance in hybrids and allopolyploid

**Figure 4:**
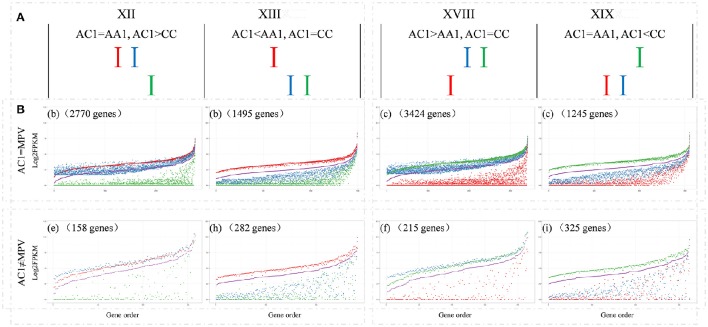
**Comparisons of the expression levels of genes showing expression level dominance in AC1. (A)** The four expression level dominance patterns, for which the gene expression level in hybrid AC1 is statistically similar to one parent but different from other parent. **(B)** The normalized expression values, measured in hybrid, the MPV and two progenitors, for genes from the four expression patterns are plotted to illustrate the expression level dominance. The y-axis represents log_2_ of the expression level (FPKM). Genes are ordered on the x-axis according to increasing FPKM of their MPV. The number of genes in each comparison is also indicated. Different colors indicate different species as described above, Red, AA1; green, CC; blue, AC1; purple, MPV.

Among those genes showing differential expression between parents, approximate 37.0% exhibited expression level dominance in AC1, for their expression levels were statistically similar to one parent but different from the other parent (9914, patterns XII+XIII+XVIII+XIX). To further depict expression level dominance, we plotted the normalized expression values, measured in hybrid, the MPV and two progenitors, for genes from the four expression patterns (Figure 4). Notably, most of the genes (8934, 90.1%; bXII+bXIII+cXVIII+cXIX) showing expression level dominance were additively expressed in hybrids, suggesting that expression level dominance was accompanied with additive expression in most cases as described by Rapp et al. (2009) and Chagué et al. (2010). The remaining 980 genes (eXII+hXIII+fXVIII+iXIX) showed differential expression between hybrid and MPV, which represented a small fraction of the total dominance genes (9.9%) but covered a large proportion of non-additively expressed genes (41.1%).

There were 4499 (45.4%, XII+XIX) genes exhibiting expression level dominance toward the A-genome parent, for which the expression values in AC1 were equal to those of the A-genome parent and higher (2928, bXII+eXII) or lower (1570, cXIX+iXIX) than those of C-genome parent. Conversely, a slightly more genes (5416, 54.6%, XIII+XVIII) were toward the C-genome parent, including 3639 genes (cXVIII+fXVIII) that were up-regulated and 1777 gene (bXIII+hXIII) that were down-regulated in the C-genome parent when compared with the A-genome parent. However, the parental bias was insignificant when we compared the number of genes from the four categories which represented four forms of expression level dominance (A-dominance: bXII, cXIX, eXII, iXIX vs. C-dominance: bXIII, cXVIII, hXIII, fXVIII; *P* > 0.05, *t*-test). As to AC2 and AACC, there were slightly more parental dominance genes than that in AC1 (12084 in AC2 and 13695 in AACC vs. 9914 in AC1), with no preference for either parental genome (Figures S4, S5; Table S3; *P* > 0.05, *t*-test).

**Figure 5:**
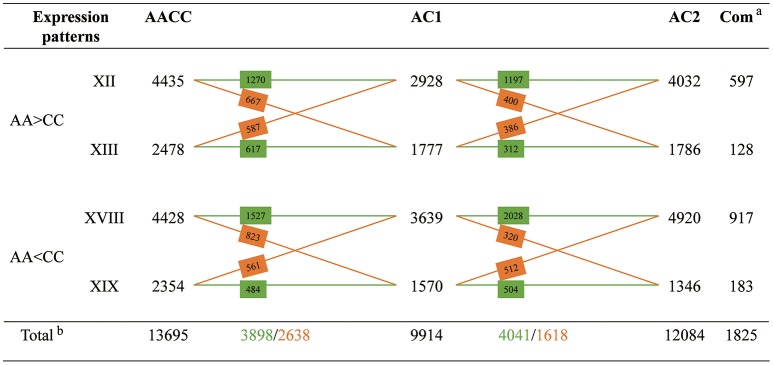
**Cross comparisons of the four different patterns of expression level dominance between synthetic hybrids**. Numbers of genes shared between different expression patterns are indicated on the cross lines. The green color indicates the number of genes which exhibiting the same pattern between two samples; The orange color indicates the number of genes which showing different expression patterns between two samples.

Comparison of the four patterns of parental dominance revealed that multitudes of genes were shared and exhibited similar patterns among the two hybrids and allopolyploid (Figure 5). The expression patterns in hybrid AC1 were more similar to hybrid AC2 than to allopolyploid AACC, with more genes in common and less reverse. Similar functional classes of genes were affected in both hybrids and allopolyploid (Table S4). We further pursued possible functions of shared genes between the two hybrids from the four expression patterns, respectively. The GO slims were almost identical in pattern XII and XVIII, for genes involved in metabolic process, translation, structural molecule activity and structural constituent of ribosome could be enriched in both patterns. In addition, we observed that most GO terms in pattern XIII and XIX were related to stress response, suggestive of high similarity in function between A-genome (patterns XII and XIX) and C-genome (patterns XIII and XVIII) dominance genes.

### Homoeolog expression bias in hybrids and allopolyploid

To estimate the extent of homoeolog expression bias as well as parental legacy on homoeolog expression, we compared their expression levels of homoeologous gene pairs between the parental diploids and their derived subgenomes in hybrids/allopolyploid (Table 1). The expression patterns observed in the parental diploids showed high conservation in their derived hybrids/allopolyploid. Most gene pairs (74.3% in AC1, 73.6% in AC2 and 69.5% in AACC, respectively) showing similar expression levels in the two parents displayed such preconditioned expression patterns between the corresponding homoeologs in the hybrids/allopolyploid. Furthermore, the parental homoeolog biases were maintained for 3.1–4.1% of all the gene pairs, with no significant bias toward either homoeolog (*P* > 0.05, Chi square test). By contrast, 4.2–8.3% gene pairs exhibiting preexisting expression bias reverted to non-differential expression in the hybrids/allopolyploid. Only a small number of genes exhibited novel bias, for genes showed differential or opposite homoeolog expression patterns in the hybrids/allopolyploid relative to their patterns in the two parents. Overall, 4367 (1186 in AC1; 1339 in AC2; 1842 in AACC) and 4379 (1292 in AC1; 1323 in AC2; 1764 in AACC) gene pairs showed homoeolog bias toward A or C genome, respectively, indicating no preference for either parental genome (*P* > 0.05, *t*-test).

**Table 1:** **Homoeolog expression bias in the two synthetic hybrids and natural allopolyploid**.

**Homoeolog expression**	**Parents^a^**	**Hybrid**	**AC1(%)^b^**	**AC2(%)**	**Common^c^**	**AACC (%)**
**PARENTAL CONDITION**						
	A = C	A = C	16962(74.3)	16676(73.6)	14392	15122(68.5)
	A > C	A > C	945(4.1)	900(4.0)	499	680(3.1)
	A < C	A < C	899(3.9)	814(3.6)	467	712(3.2)
**NO BIAS IN HYBRID**						
	A > C	A = C	1884(8.3)	1739(7.7)	686	1915(6.5)
	A < C	A = C	1512(6.6)	1575(7.0)	771	1445(4.2)
**NOVEL BIAS IN HYBRID**						
	A = C	A > C	188(0.8)	356(1.6)	33	933(4.2)
	A = C	A < C	302(1.3)	407(1.8)	47	823(3.7)
	A > C	A < C	91(0.4)	102(0.5)	9	229(1.0)
	A < C	A > C	53(0.2)	83(0.4)	9	229(1.0)
Overall A-bias in hybrid			1186(5.2)	1339(5.9)	673	1842(8.3)
Overall C-bias in hybrid			1292(5.7)	1323(5.8)	667	1764(8.0)
Total number of genes			22836	22652	16913	22088

a *A = C represented equal expression between A and C homoeolog; A > C and A < C represented A and C homoeolog expression bias, respectively*.

b *Calculated by dividing the total number of commonly expressed genes in each contrast*.

c *Commonly expressed genes among the parents and two hybrids in each expression patterns*.

In addition, nearly half of the genes showing homolog bias were shared in the hybrids, including 673 of A-bias and 667 of C-bias gene pairs. GO analysis of the homoeolog- expression-bias gene pairs shared between the two hybrids AC1 and AC2, revealed that those involved in structural molecule activity, generation of precursor metabolites and energy and ribosome were enriched in A-bias patterns, whereas those involved in oxidoreductase activity, translation, and cytoplasm were enriched in C-bias patterns (Table S5). Analysis of those gene pairs which specifically expressed and non-shared in the two hybrids was also performed and the GO terms were provided (Table S5).

### Verification of gene expression by qRT-PCR

To confirm the gene expression data above, a set of gene-specific primers were designed for quantitative RT-PCR assays (Table S6). The relative transcript levels were then compared with those of RNA-seq data (average FPKM value from two replications). For 19 out of the 22 comparisons, qRT-PCR analysis revealed the same expression trends as the RNA-seq data, despite some quantitative differences, confirming the reliability of RNA-seq data (Figure S6).

## Discussion

### Non-additive expressions in synthetic and natural *B. napus*

As noticed in various studies, the expression levels of genes in the allopolyploids were not simply the average of that of the two parents, while many of the observed gene expression changes were non-additive (Wang et al., 2006; Chagué et al., 2010; Yoo et al., 2013; Zhao et al., 2013; Jiang et al., 2015). Based on analysis of the transcriptome data from synthetic *B. napus* hybrids and their parents, we found that non-additive expression occurred immediately after the two genomes merge in hybrids, for many genes in the hybrids displayed expression divergence from their *in silico* MPVs (Figure 2). The percentage of non-additive genes in *B. napus* hybrids (4.5–5.8%) was similar to that in *Arabidopsis* allotetraploids (5.2–5.6%; Wang et al., 2006), wheat allohexaploids (6.6%; Chagué et al., 2010), cotton hybrid (10.8%; Yoo et al., 2013), and *Brassica* allohexaploid (7.8%; Zhao et al., 2013). Due to the lack of the extant diploid that was truly representative of the original C-genome parent of the natural *B. napus*, comparisons may not be entirely appropriate. However, the higher percentage of non-additive genes (16.0%) in natural *B. napus* was consistent with the higher 16.3–18.3% in natural cotton allotetraploid than 10.8% in synthetic hybrid (Yoo et al., 2013). The overall distribution of most non-additively expressed genes along the chromosomes was largey random, but large regions of down-regulated genes appeared along some chromosomes (Figure S1). However, the generality of this conclusion needed further investigation, considering the possible deviation of low number of non-additively expressed genes in those chromosomes.

### Balanced expression level dominance in the synthetic hybrids

Expression level dominance, for which the expression level of genes in the allopolyploid mimicked the expression level of one parent, irrespective of whether genes were up or down-regulated relative to the other parent, was first advocated by Rapp et al. (2009). Then expression level dominance was further demonstrated in many different allopolyploids, including cotton (Flagel and Wendel, 2010), *Spartina* (Chelaifa et al., 2010), *coffea* (Bardil et al., 2011), wheat (Chagué et al., 2010; Li et al., 2014), and others. In consistence with those previous findings, we found that a marked number of genes displayed expression level dominance in the synthetic *B. napus* hybrids, and majority of them were additively expressed as well (Figure 3 and Figure S4; Table S3). However, the synthetic hybrids exhibited balanced expression level dominance, with no preference for either parental genome. It is worthwhile to study the gene expression of other tissues besides young leaves from these hybrids, as expression level dominance was tissue-specific and temperature-dependent as described in cotton (Yoo et al., 2013) and *coffea* (Bardil et al., 2011).

Besides the approximately equivalence in number genes, we also observed high similarity in function between A and C-genome parental dominance genes (Table S4). Those genes were enriched in metabolic process, translation, structural molecule activity, structural constituent of ribosome, as well as stress response in both A and C-genome dominance patterns, suggesting that those housekeeping or stress response genes might be inclined to keep the expression levels from one of their parents. Together, the equivalence in number and similarity in function indicated that there might be a balance of parental expression level dominance after the merger of two genomes in synthetic hybrids.

### Parental legacy of homoeolog expression bias in the synthetic hybrids and natural allopolyploid

The phenomenon of homoeolog expression bias in allopolyploids has been described in several studies using various methods and techniques (Adams et al., 2003; Chelaifa et al., 2010; Flagel and Wendel, 2010; Yoo et al., 2013; Chalhoub et al., 2014; Li et al., 2014). Owing to the advantages of new techniques, genome-wide comparison of homoeolog expressions within allopolyploids, or between the two parental diploids was available using RNA-seq (Yoo et al., 2013). Moreover, the availability of reference genome of *B. napus* (Chalhoub et al., 2014) provided prior information to distinguish the homoeologous gene pairs and to study the homoeolog expression bias in our synthetic hybrids and natural allopolyploid. Comparison of homoeolog expressions bias demonstrated that the expression patterns observed in the parental diploids were often conserved in their derived hybrids and allopolyploid, especially when the two homoeologs were expressed at similar expression level (Table 1). Similarly, recent studies of cotton and wheat also showed that parental patterns of homoeolog expressions bias were generally maintained at higher level in natural allopolyploids relative to hybrids (Yoo et al., 2013; Li et al., 2014). Furthermore, majority of gene pairs showing homoeolog expressions bias in hybrids also preexisted, indicating that homoeolog expressions bias could be vertically inherited from diploid parents in most cases. In accordance with expression level dominance, the overall homoeolog expressions bias in *B. napus* hybrids and allopolyploid were also balanced, which mean that there was no significant bias toward either A or C subgenome.

### High conservation of gene expressions after genome merger

Because the precise parental genotypes of extant *B. napus* were unknown after its formation of several thousands of years, the extracted *B. rapa* from *B. napus* (Tu et al., 2010) was an excellent material to investigate the gene expression changes during the allopolyploidization process. Only a small fraction of genes exhibited differential expression between the extracted (AA1) and natural (AA2) *B. rapa*, and approximately a quarter of them were shared in their derived hybrids (Figure 2). Previous studies in synthetic allohexaploid wheat (AABBDD) parented by the extracted tetraploid wheat (AABB) showed that the majority of genes were additive expression (Chelaifa et al., 2013; Zhang et al., 2014). The prevalence of gene expression additivity in our hybrids was in line with the observations in wheat, while slightly fewer non-additively expressed genes existed in the hybrid AC1 than the hybrid AC2. Moreover, comparisons revealed that the majority of additively expressed genes (91.7%) and a high proportion of non-additively expressed genes (26.9%) were shared between the two hybrids (Figure S2), comparable with higher proportion (34.5–47%) of shared genes in resynthesized *Arabidopsis* and wheat allopolyploids (Wang et al., 2006; Chagué et al., 2010). In addition, nearly one half of genes/gene pair showing expression level dominance and homoeolog expression bias were shared between the two hybrids (Figure 5; Table 1), revealing the substantial conservation of gene expressions. Similarly, a slightly fewer genes/gene pair were found in AC1 than AC2 despite the balanced expression observed in both hybrids. These results suggested that the A genome experiencing allopolyploidy responded less to the repeated genome merger and was more compatible in the interaction with C genome than the new comer from natural *B. rapa*. The similar and conserved patterns of gene expressions between the extracted and natural *B. rapa*, as well as between their derived hybrids also showed that the structural alterations of A genome caused by frequent homeologous exchanges in *B. napus* did not disturbed much its performance in new hybrids (Chalhoub et al., 2014).

### Balanced maintenance during domestication at allotetraploid level

Comparative genomics studies showed that homoeologous exchanges (HEs) were frequent between natural *B. napus* subgenomes and ranged from SNPs to chromosome segments (Chalhoub et al., 2014). The artificial selection of favorable agronomic traits, such as oil biosynthesis, disease resistance, and flowering, could lead to preferred preservation of HEs containing these genes during the domestication of *B. napus*. Such gradual and sequential accumulation of genetic changes could trigger extensive changes in gene expression, as described in our study (Figure 2D). A higher proportion of differentially expressed genes between natural allotetraploid and hybrid indicated that many genes have diverged their expression over evolutionary time. Furthermore, the number of transgressively expressed genes increased sharply, which supported the hypothesis that transgressive expression evolved *de novo* in the allotetraploid (Flagel and Wendel, 2010). Although, more differential and transgressive expression changes likely occurred during domestication than those caused by genome merge, the number of genes/gene pairs showing expression level dominance and homoeolog expression bias increased not as obviously as above (Figure 5 and Table 1). Unlike in other allopolyploids exhibiting different magnitude of imbalance or reverse direction, the balanced expression between the two divergent but homoeologous genomes was established soon after the incipient genome merger and maintained in magnitude during domestication at allotetraploid level.

Though the underlying mechanisms of expression level dominance and homoeolog expressions bias were still not completely understood, two hypotheses could be put forward. In *Arabidopsis* allopolyploid, *cis* and *trans*-regulatory was associated with chromatin modifications and has different effect on allelic expression, divergence of them between progenitor species determined gene expression novelty in allopolyploids (Shi et al., 2012). Recent studies in mesohexaploid *B. rapa* and nascent allohexaploid wheat found higher density of transposon-derived siRNA target at recessive subgenome, and siRNA-mediated silencing of transposons near genes might causes position-effect down-regulation of homoeologs, account for biased repression of subgenome (Li et al., 2014; Woodhouse et al., 2014). We assumed that significantly lower TE content (34.8% vs. 64.8%), relatively less asymmetric subgenome (314 Mb A and 525 Mb C subgenome in *B. napus*; 1477 Mb A and 831 Mb D subgenome in cotton), as well as shorter history of asymmetric evolution (~7500YA vs. 1-2MYA), might account for the absence of significant bias toward either subgenome in *B. napus* as compared with cotton (Chalhoub et al., 2014; Zhang T. et al., 2015).

Homoeolog expression bias and especially expression level dominance, as two genome-wide consequences of allopolyploidization, have been documented in only a handful of allopolyploids and barely explored in *B. napus*. Herein, we studied these two phenomena in synthetic and natural *B. napus* and detailed those changes during short- and long-term evolutionary process. Expression level dominance and homoeolog expressions bias were balanced at the initial stage of genome merger, and such balance were maintained during the domestication of *B. napus*. We also found that the extracted *B. rapa* appeared more compatible for hybridization than the natural *B. rapa* despite the overall patterns of gene expression were highly conserved between their derived hybrids. Our study provides novel insights into the architecture of gene expression during the hybridization and domestication in *B. napus* and the effect of A genome with different origins. Considering that these two phenomena are tissue-specific and environmental-dependent and the mechanisms remains unclear, further studies using various tissues and environmental conditions, as well as new methods (such as siRNA and methylation analysis) are still needed to understand the role of epigenetic modulation and their interconnections.

## Author contributions

ZL and YS: Conceived the study; CT, BZ, XG: Participated in sample preparations for RNA-seq; QP: Helped to analyze the data; DZ and ZL: Analyzed the data and wrote the manuscript; All authors have read and approved the final manuscript.

## Data archiving

Short reads supporting the results of this article are available in GenBank SRA under accession ID PRJNA307139. Other supporting data are included within the article and its additional files.

### Conflict of interest statement

The authors declare that the research was conducted in the absence of any commercial or financial relationships that could be construed as a potential conflict of interest.

## Supplementary material

Figure S1 **The overall distribution of non-additively expressed genes along some chromosomes in AC1**. The horizontal axis represents the physical location of each chromosome. The vertical axis represents the normalized fold change between AC1 and MPV. The red and blue points indicate the genes over-expressed or under-expressed in AC1 relative to MPV, respectively.

Figure S2 **Cross comparisons of sets of additively and non-additively expressed genes in two synthetic hybrids**. Numbers of genes shared between different expression patterns are indicated on the cross lines.

Figure S3 **Cross comparisons of sets of differentially and non-differentially expressed genes revealed between synthetic hybrids and their relative A genome progenitors**. Numbers of genes shared between different expression patterns are indicated on the cross lines.

Figure S4 **Global classification of gene expressions among hybrid AC2 and its progenitors**. Genes based on differentially or non-differentially among the hybrid, diploid progenitors, and MPV, were further classified into **(A)** 19 possible expression patterns (I—XIX; AA2 vs. CC, AA2 vs. AC2, AC2 vs. CC) and **(B)** 9 major expression categories (a–i; AC2 vs. MPV, AA2 vs. CC). Red, green and blue bars represent the confidence intervals of expression level observed in A and C genome progenitors and hybrid, respectively. A gene is differentially expressed between two samples when their confidence intervals (bars) do not overlap whereas it is equal when they do overlap. The x-axis represents the 19 possible expression patterns. The y-axis represents gene count. The number of genes for each expression pattern in each of expression categories is also indicated.

Figure S5 **Comparison of the expression levels of genes showing expression level dominance in AC2. (A)** The four expression level dominance patterns, for which the gene expression level in hybrid AC2 is statistically similar to one parent but different from the other parent. **(B)** The normalized expression values, measured in hybrid, the MPV and two progenitors, for genes from the four expression patterns are plotted to illustrate the expression level dominance. The y-axis represents log_2_ of the expression level (FPKM). Genes are orders on the x-axis according to increasing FPKM of their MPV. The number of genes in each comparison is also indicated. Different colors indicate different species as described above, Red, AA2; green, CC; blue, AC2; purple, MPV.

Figure S6 **qRT-PCR confirmation of the differentially expressed genes**. Columns and bars represent the means and standard error from qRT-PCR (*n* = 3), respectively. The average gene expression levels from two RNA-Seq replications are indicated on the bottom of each gene.

Table S1 **GO analysis of commonly regulated genes in both AA1 and AC1 as comparing with AA2 and AC2**.

Table S2 **GO analysis of non-additively expressed genes in the two hybrids**.

Table S3 **Global classification of gene expression among allopolyploid and its progenitors**.

Table S4 **GO analysis of genes showing expression level dominance in two hybrids**.

Table S5 **GO analysis of genes showing homoeolog expression bias in two hybrids**.

Table S6 **The corresponding primers of qRT-PCR**.
