# Supplementary figures and images for: Genome-Wide Gene Expressions Respond Differently to A-subgenome Origins in Brassica napus Synthetic Hybrids and Natural Allotetraploid
Source: Front Plant Sci. 2016 Oct 13;7:1508. doi: 10.3389/fpls.2016.01508 (PMC5061818; doi:10.3389/fpls.2016.01508)

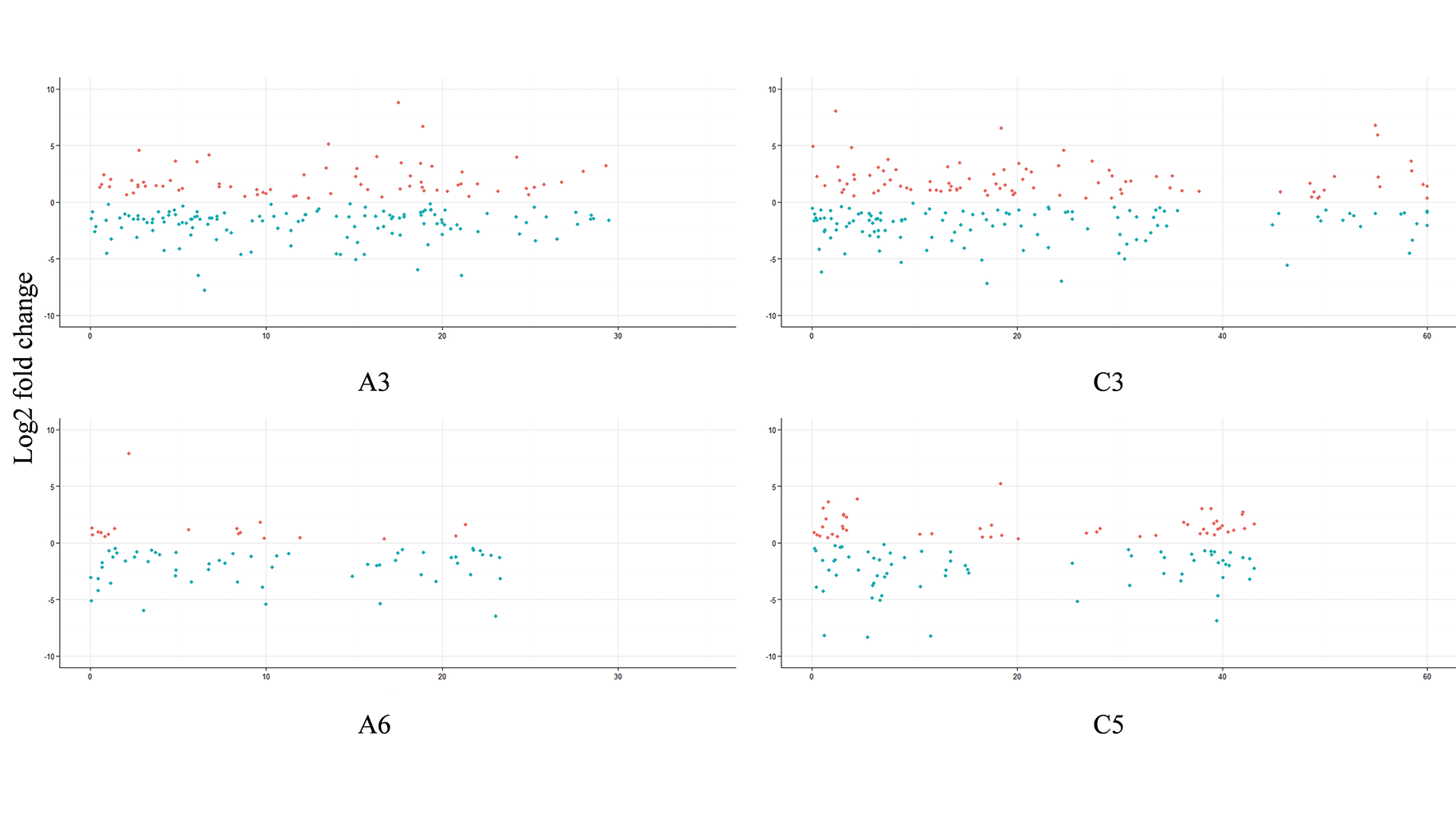

Supplement: Figure S1 — The overall distribution of non-additively expressed genes along some chromosomes in AC1. The horizontal axis represents the physical location of each chromosome. The vertical axis represents the normalized fold change between AC1 and MPV. The red and blue points indicate the genes over-expressed or under-expressed in AC1 relative to MPV, respectively. [file Image1.TIF]

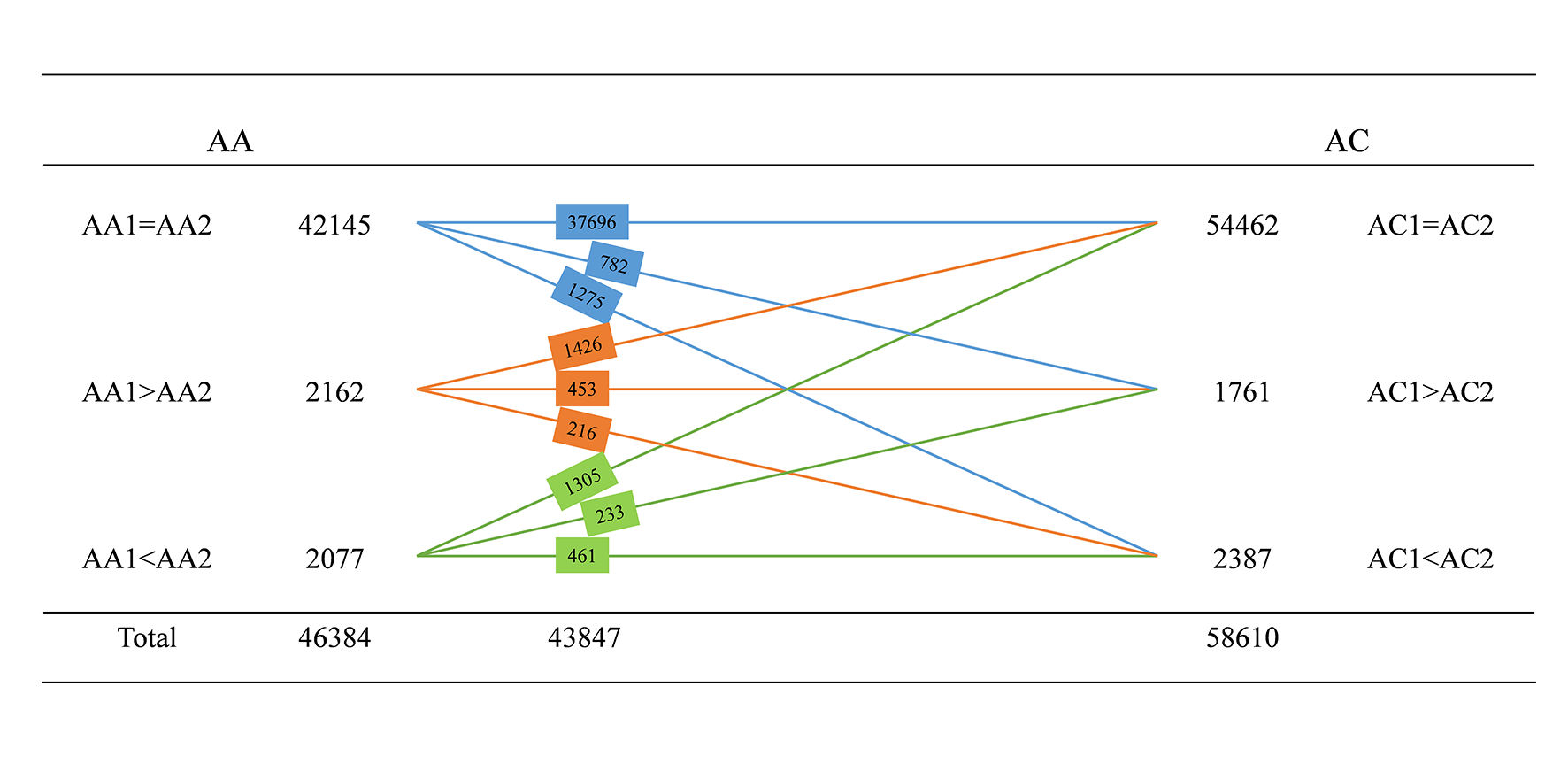

Supplement: Figure S2 — Cross comparisons of sets of additively and non-additively expressed genes in two synthetic hybrids. Numbers of genes shared between different expression patterns are indicated on the cross lines. [file Image2.TIF]

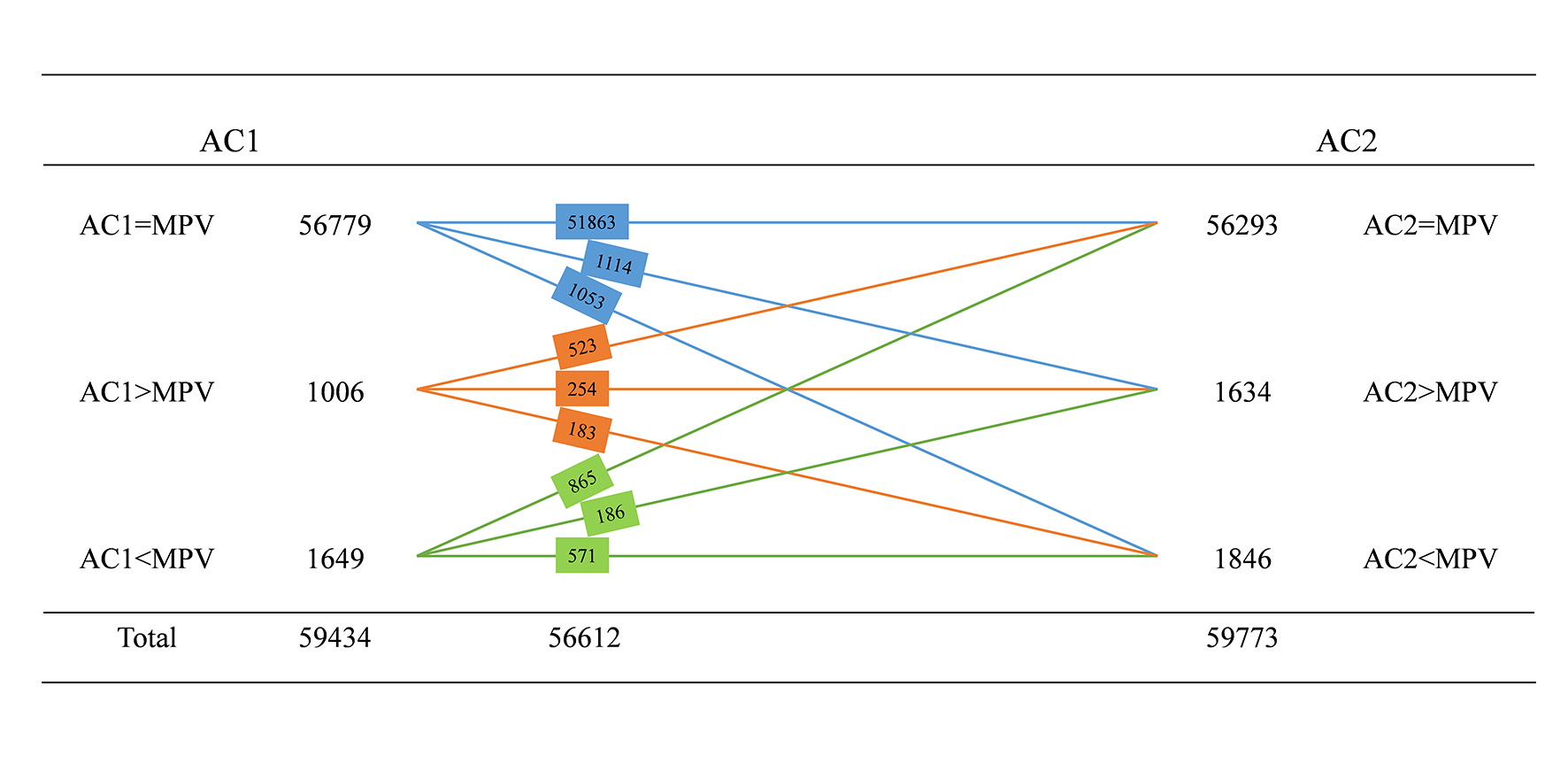

Supplement: Figure S3 — Cross comparisons of sets of differentially and non-differentially expressed genes revealed between synthetic hybrids and their relative A genome progenitors. Numbers of genes shared between different expression patterns are indicated on the cross lines. [file Image3.TIF]

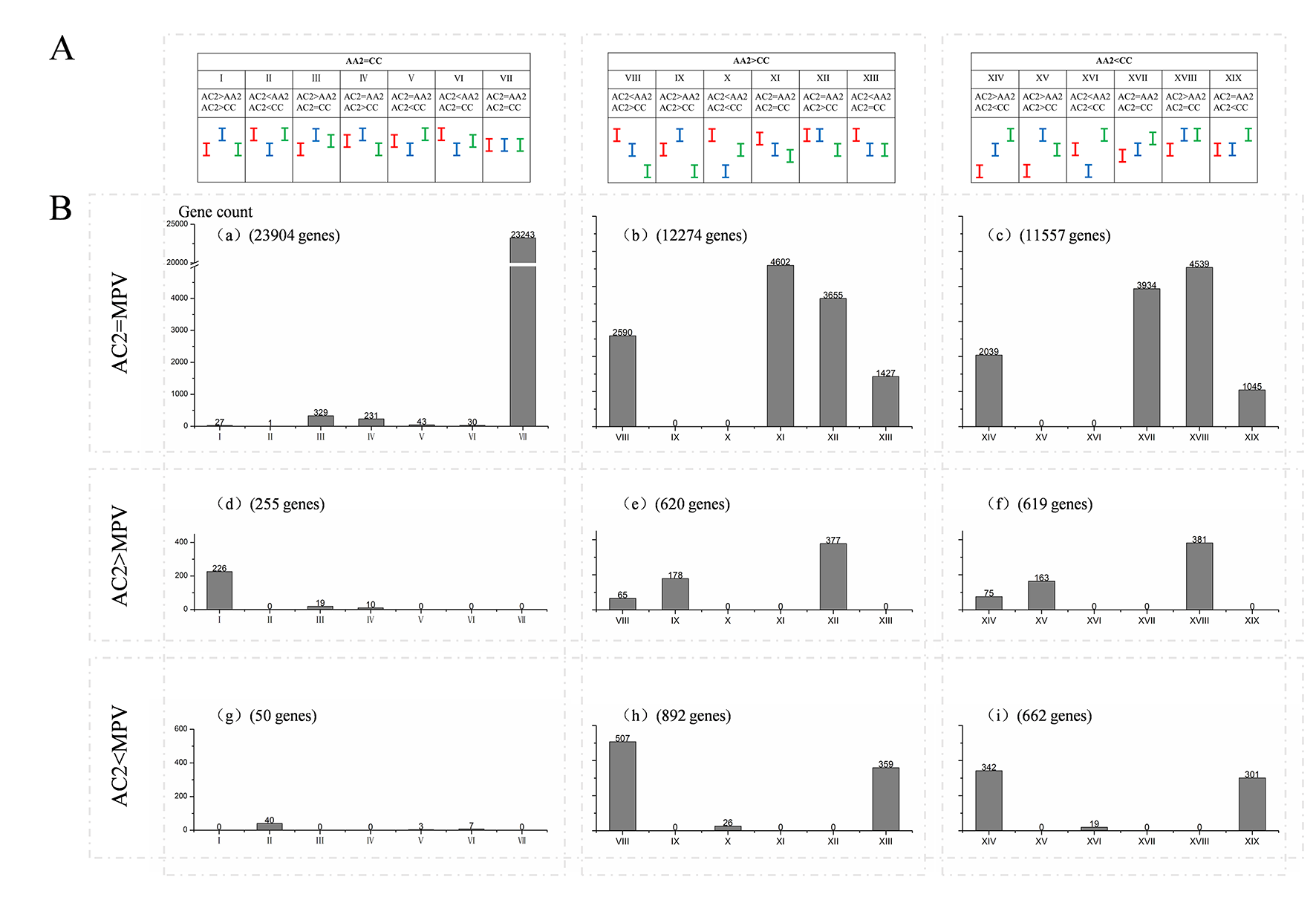

Supplement: Figure S4 — Global classification of gene expressions among hybrid AC2 and its progenitors. Genes based on differentially or non-differentially among the hybrid, diploid progenitors, and MPV, were further classified into (A) 19 possible expression patterns (I—XIX; AA2 vs. CC, AA2 vs. AC2, AC2 vs. CC) and (B) 9 major expression categories (a–i; AC2 vs. MPV, AA2 vs. CC). Red, green and blue bars represent the confidence intervals of expression level observed in A and C genome progenitors and hybrid, respectively. A gene is differentially expressed between two samples when their confidence intervals (bars) do not overlap whereas it is equal when they do overlap. The x-axis represents the 19 possible expression patterns. The y-axis represents gene count. The number of genes for each expression pattern in each of expression categories is also indicated. [file Image4.TIF]

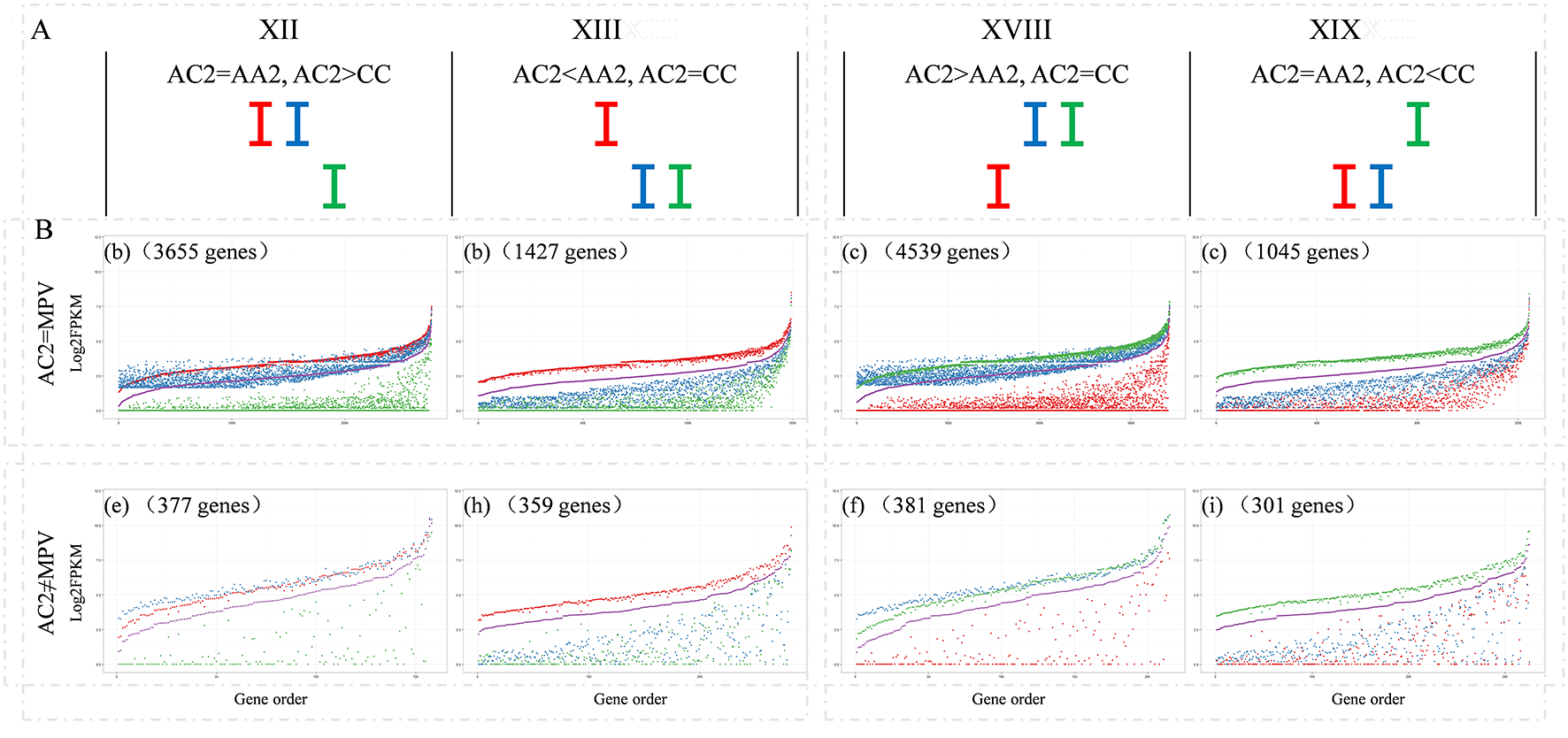

Supplement: Figure S5 — Comparison of the expression levels of genes showing expression level dominance in AC2. (A) The four expression level dominance patterns, for which the gene expression level in hybrid AC2 is statistically similar to one parent but different from the other parent. (B) The normalized expression values, measured in hybrid, the MPV and two progenitors, for genes from the four expression patterns are plotted to illustrate the expression level dominance. The y-axis represents log2 of the expression level (FPKM). Genes are orders on the x-axis according to increasing FPKM of their MPV. The number of genes in each comparison is also indicated. Different colors indicate different species as described above, Red, AA2; green, CC; blue, AC2; purple, MPV. [file Image5.TIF]

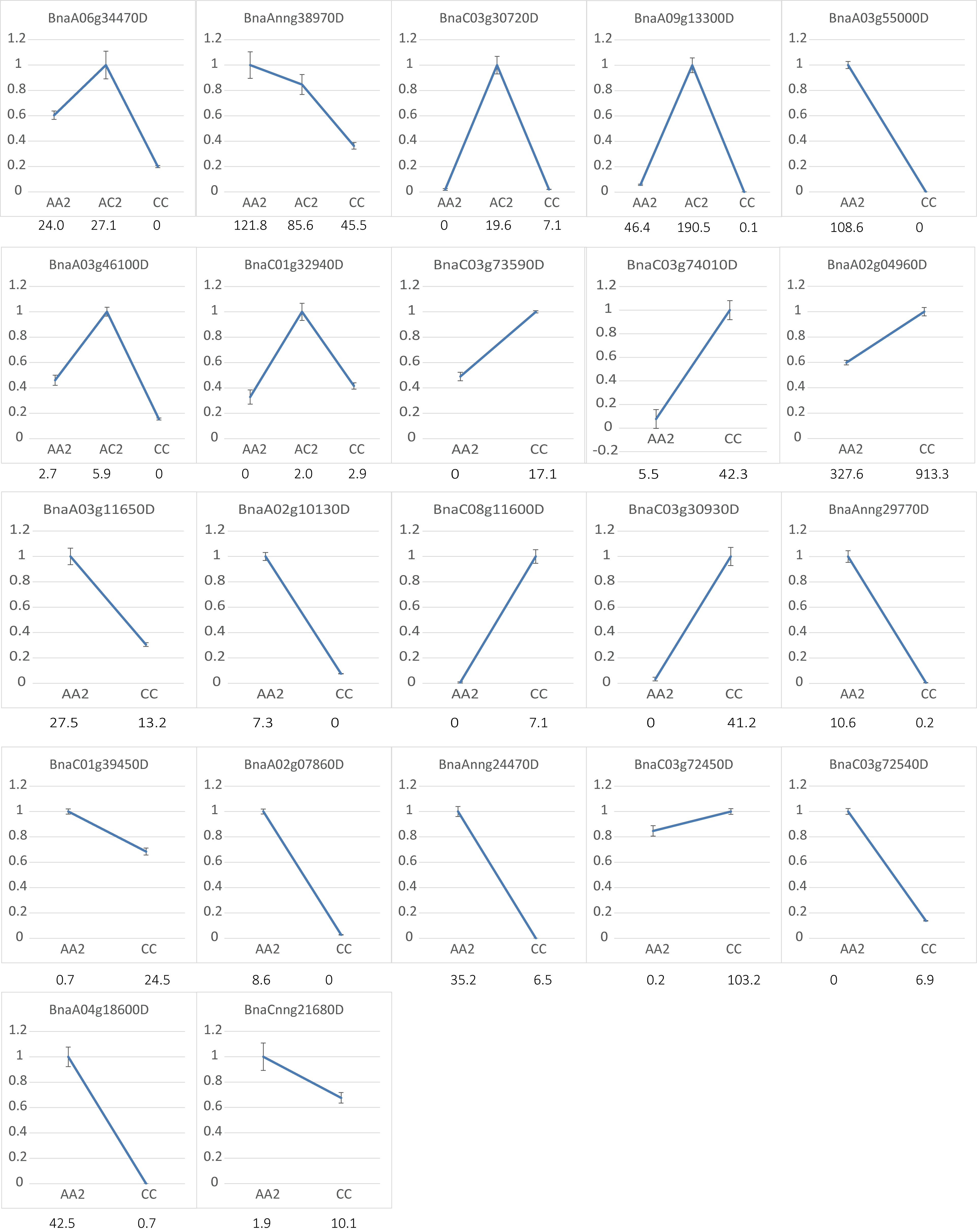

Supplement: Figure S6 — qRT-PCR confirmation of the differentially expressed genes. Columns and bars represent the means and standard error from qRT-PCR (n = 3), respectively. The average gene expression levels from two RNA-Seq replications are indicated on the bottom of each gene. [file Image6.tif]
