# Supplementary material for: Genome-Wide Gene Expressions Respond Differently to A-subgenome Origins in Brassica napus Synthetic Hybrids and Natural Allotetraploid
Source: Front Plant Sci. 2016 Oct 13;7:1508. doi: 10.3389/fpls.2016.01508 (PMC5061818; doi:10.3389/fpls.2016.01508)
Supplement: Table S1 — GO analysis of commonly regulated genes in both AA1 and AC1 as comparing with AA2 and AC2. [file Table1.DOC]

| **Table S1** **GO analysis of commonly regulated genes in both AA1 and AC1 as comparing with AA2 and AC2.** | | | |
| --- | --- | --- | --- |
|  |  |  | |
| GO-slim | Gene count | | P value |
| up-regulated; AA1>AA2 and AC1>AC2 |  | |  |
| Molecular Function |  | |  |
| catalytic activity | 47 | | 2.02E-03 |
| carbohydrate transmembrane transporter activity | 9 | | 1.02E-03 |
| transferase activity | 6 | | 7.02E-06 |
| kinase activity | 2 | | 3.22E-03 |
| protein kinase activity | 1 | | 1.96E-02 |
|  |  | |  |
| Biological Process |  | |  |
| metabolic process | 68 | | 2.79E-03 |
| primary metabolic process | 54 | | 1.06E-02 |
| carbohydrate transport | 9 | | 1.36E-02 |
| nucleobase-containing compound metabolic process | 8 | | 4.39E-04 |
| cellular protein modification process | 5 | | 4.31E-03 |
| protein phosphorylation | 2 | | 2.43E-02 |
|  |  | |  |
| Cellular Component |  | |  |
| integral to membrane | 9 | | 4.72E-05 |
| plasma membrane | 8 | | 1.64E-02 |
| cell junction | 5 | | 4.42E-04 |
| tubulin complex | 4 | | 2.15E-03 |
|  |  | |  |
| down-regulated; AA1<AA2 and AC1<AC2 |  | |  |
| Molecular Function |  | |  |
| structural molecule activity | 39 | | 3.75E-11 |
| oxidoreductase activity | 30 | | 1.79E-02 |
| structural constituent of ribosome | 26 | | 1.90E-12 |
| isomerase activity | 12 | | 2.16E-02 |
| translation elongation factor activity | 8 | | 5.24E-03 |
|  |  | |  |
| Biological Process |  | |  |
| protein metabolic process | 69 | | 2.19E-03 |
| translation | 39 | | 2.57E-12 |
|  |  | |  |
| Cellular Component |  | |  |
| ribonucleoprotein complex | 10 | | 1.87E-02 |
| ribosome | 7 | | 1.37E-03 |
| cytosol | 6 | | 2.35E-03 |
