# Supplementary material for: Genome-Wide Gene Expressions Respond Differently to A-subgenome Origins in Brassica napus Synthetic Hybrids and Natural Allotetraploid
Source: Front Plant Sci. 2016 Oct 13;7:1508. doi: 10.3389/fpls.2016.01508 (PMC5061818; doi:10.3389/fpls.2016.01508)
Supplement: Table S2 — GO analysis of non-additively expressed genes in the two hybrids. [file Table2.DOC]

**Table S2 GO analysis of nonadditively expressed genes in the two hybrids.**

**GO-slims nonadditively expressed gene showing up-regulation in AC1**

| GO-Slim | Gene count | P value |
| --- | --- | --- |
| Molecular Function |  |  |
| carbohydrate transmembrane transporter activity | 10 | 1.61E-02 |
| structural constituent of ribosome | 17 | 4.98E-02 |
| oxidoreductase activity | 46 | 1.29E-02 |
| transferase activity | 30 | 4.18E-02 |
| binding | 41 | 3.09E-04 |
| receptor activity | 2 | 1.75E-02 |
| transmembrane receptor protein kinase activity | 1 | 1.46E-02 |
|  |  |  |
| Biological Process |  |  |
| sulfur compound metabolic process | 12 | 1.56E-02 |
| homeostatic process | 12 | 1.98E-02 |
| cellular protein modification process | 17 | 2.79E-02 |
| nucleobase-containing compound metabolic process | 18 | 7.76E-06 |
| intracellular protein transport | 8 | 2.19E-02 |
| protein transport | 8 | 1.16E-02 |
| RNA metabolic process | 8 | 9.25E-04 |
|  |  |  |
| Cellular Component |  |  |
| cell junction | 5 | 6.40E-03 |
| ribosome | 8 | 8.01E-03 |
| integral to membrane | 8 | 2.12E-02 |
| plasma membrane | 10 | 4.80E-02 |

GO-slims nonadditively expressed gene showing down-regulation in AC1

| GO-Slim | Gene count | P value |
| --- | --- | --- |
| Molecular Function |  |  |
| cysteine-type endopeptidase inhibitor activity | 4 | 1.80E-02 |
| receptor binding | 19 | 4.45E-05 |
| translation elongation factor activity | 15 | 1.05E-03 |
| calmodulin binding | 15 | 2.26E-02 |
| oxidoreductase activity | 82 | 7.87E-06 |
| structural molecule activity | 51 | 2.10E-02 |
| transporter activity | 28 | 3.62E-02 |
| transmembrane transporter activity | 26 | 3.36E-02 |
| kinase activity | 18 | 1.68E-04 |
| protein kinase activity | 13 | 3.99E-03 |
| transmembrane receptor protein kinase activity | 4 | 2.18E-03 |
| receptor activity | 4 | 1.34E-04 |
|  |  |  |
| Biological Process |  |  |
| protein folding | 26 | 6.51E-06 |
| response to toxic substance | 20 | 1.46E-02 |
| response to stress | 39 | 2.03E-03 |
| response to stimulus | 69 | 1.63E-03 |
| transport | 55 | 4.51E-02 |
| localization | 56 | 3.63E-02 |
| cellular protein modification process | 33 | 2.67E-03 |
| nucleobase-containing compound metabolic process | 43 | 8.64E-06 |
| RNA metabolic process | 20 | 3.73E-04 |
| protein phosphorylation | 15 | 6.52E-04 |
| intracellular protein transport | 14 | 1.21E-04 |
| protein transport | 14 | 4.13E-05 |
| DNA metabolic process | 3 | 3.20E-02 |
|  |  |  |
| Cellular Component |  |  |
| macromolecular complex | 13 | 1.97E-02 |
| protein complex | 5 | 9.40E-04 |

GO-slims nonadditively expressed gene showing up-regulation in AC2

| GO-Slim | Gene count | P value |
| --- | --- | --- |
| Molecular Function |  |  |
| structural constituent of ribosome | 42 | 2.55E-11 |
| structural molecule activity | 71 | 5.47E-10 |
| isomerase activity | 23 | 2.31E-02 |
| oxidoreductase activity | 84 | 3.00E-07 |
| protein binding | 23 | 3.68E-02 |
|  |  |  |
| Biological Process |  |  |
| sulfur compound metabolic process | 20 | 8.17E-05 |
| translation | 67 | 6.44E-10 |
| monosaccharide metabolic process | 24 | 9.76E-03 |
| carbohydrate metabolic process | 56 | 2.58E-02 |
| protein metabolic process | 165 | 4.21E-03 |
| metabolic process | 353 | 5.43E-03 |
| nucleobase-containing compound metabolic process | 52 | 1.45E-02 |
| protein transport | 20 | 3.17E-02 |
| protein phosphorylation | 17 | 1.12E-02 |
| RNA metabolic process | 17 | 6.93E-05 |
| transcription, DNA-dependent | 9 | 5.82E-03 |
| transcription from RNA polymerase II promoter | 8 | 6.90E-03 |
| regulation of nucleobase-containing compound metabolic process | 4 | 9.44E-03 |
| regulation of transcription from RNA polymerase II promoter | 3 | 2.00E-02 |
| vesicle-mediated transport | 2 | 6.16E-04 |
| catabolic process | 1 | 1.63E-02 |
|  |  |  |
| Cellular Component |  |  |
| ribosome | 14 | 2.02E-05 |
| cytosol | 9 | 6.51E-03 |

GO-slims nonadditively expressed gene showing down-regulation in AC2

| GO-Slim | Gene count | P value |
| --- | --- | --- |
| Molecular Function |  |  |
| carbohydrate transmembrane transporter activity | 16 | 1.84E-03 |
| binding | 107 | 1.74E-02 |
| catalytic activity | 182 | 5.29E-08 |
| hydrolase activity | 50 | 7.95E-03 |
| RNA binding | 20 | 6.68E-03 |
| transferase activity | 45 | 5.22E-09 |
| kinase activity | 18 | 3.41E-06 |
| protein kinase activity | 13 | 2.26E-04 |
| receptor activity | 7 | 1.12E-03 |
| transmembrane receptor protein kinase activity | 4 | 2.69E-04 |
|  |  |  |
| Biological Process |  |  |
| carbohydrate transport | 17 | 2.75E-02 |
| ion transport | 52 | 3.44E-04 |
| metabolic process | 282 | 1.42E-03 |
| primary metabolic process | 214 | 6.31E-05 |
| cellular process | 114 | 2.18E-02 |
| RNA metabolic process | 29 | 1.43E-02 |
| nucleobase-containing compound metabolic process | 49 | 1.98E-06 |
| phosphate-containing compound metabolic process | 13 | 2.61E-02 |
| cellular protein modification process | 27 | 1.60E-07 |
| protein phosphorylation | 17 | 1.81E-04 |
| intracellular protein transport | 16 | 3.15E-05 |
| protein transport | 16 | 9.46E-06 |
|  |  |  |
| Cellular Component |  |  |
| cell junction | 6 | 1.87E-02 |
| integral to membrane | 13 | 2.73E-03 |
| protein complex | 9 | 1.57E-02 |
