# Supplementary material for: Genome-Wide Gene Expressions Respond Differently to A-subgenome Origins in Brassica napus Synthetic Hybrids and Natural Allotetraploid
Source: Front Plant Sci. 2016 Oct 13;7:1508. doi: 10.3389/fpls.2016.01508 (PMC5061818; doi:10.3389/fpls.2016.01508)
Supplement: Table S3 — Global classification of gene expression among allopolyploid and its progenitors. [file Table3.PDF]

Table S3 Global classification of gene expression among allopolyploid and its progenitors.

Expression patterns	AA1=CC							AA1>CC						AA1<CC					
	Ⅰ	Ⅱ	Ⅲ	Ⅳ	Ⅴ	Ⅵ	Ⅶ	Ⅷ	Ⅸ	Ⅹ	Ⅺ	Ⅻ	XIII	XIV	XV	XVI	XVII	XVIII	XIX
AACC=MPV	54	2	764	621	153	73	19436	1774	0	0	3063	3594	1565	1365	0	0	2559	3691	1389
AACC>MPV	1356	0	98	72	0	0	0	37	876	0	0	841	0	31	858	0	0	737	0
AACC<MPV	0	164	0	0	25	11	0	645	0	117	0	0	913	528	0	181	0	0	965
Total	1410	166	862	693	178	84	19436	2456	876	117	3063	4435	2478	1924	858	181	2559	4428	2354
