# Supplementary material for: Genome-Wide Gene Expressions Respond Differently to A-subgenome Origins in Brassica napus Synthetic Hybrids and Natural Allotetraploid
Source: Front Plant Sci. 2016 Oct 13;7:1508. doi: 10.3389/fpls.2016.01508 (PMC5061818; doi:10.3389/fpls.2016.01508)
Supplement: Table S4 — GO analysis of genes showing expression level dominance in two hybrids. [file Table4.DOC]

**Table S4 GO analysis of genes showing expression level dominance in two hybrids.**

GO-terms of A genome dominance genes shared among hybrids and allopolyploid

| GO | Gene count | P value |
| --- | --- | --- |
| GO biological process complete |  |  |
| immune system process (GO:0002376) | 35 | 8.46E-03 |
| nucleotide metabolic process (GO:0009117) | 31 | 3.72E-02 |
| nucleoside phosphate metabolic process (GO:0006753) | 31 | 3.84E-02 |
| carbohydrate derivative metabolic process (GO:1901135) | 47 | 2.44E-02 |
| response to oxygen-containing compound (GO:1901700) | 69 | 4.46E-03 |
| organonitrogen compound metabolic process (GO:1901564) | 73 | 3.50E-03 |
| phosphorus metabolic process (GO:0006793) | 71 | 2.14E-02 |
| phosphate-containing compound metabolic process (GO:0006796) | 68 | 4.56E-02 |
| response to organic substance (GO:0010033) | 77 | 4.00E-02 |
| single-organism biosynthetic process (GO:0044711) | 90 | 1.11E-02 |
| cellular protein metabolic process (GO:0044267) | 109 | 1.01E-03 |
| small molecule metabolic process (GO:0044281) | 80 | 4.62E-02 |
| response to chemical (GO:0042221) | 98 | 2.99E-02 |
| protein metabolic process (GO:0019538) | 115 | 8.38E-03 |
| response to stress (GO:0006950) | 107 | 2.02E-02 |
| cellular metabolic process (GO:0044237) | 235 | 1.81E-04 |
| single-organism cellular process (GO:0044763) | 209 | 8.57E-03 |
| cellular process (GO:0009987) | 292 | 6.61E-04 |
| metabolic process (GO:0008152) | 265 | 3.46E-02 |
|  |  |  |
| GO cellular component complete |  |  |
| chloroplast stroma (GO:0009570) | 31 | 1.99E-05 |
| plastid stroma (GO:0009532) | 31 | 4.01E-05 |
| chloroplast part (GO:0044434) | 53 | 8.54E-08 |
| plastid part (GO:0044435) | 53 | 1.62E-07 |
| organelle subcompartment (GO:0031984) | 29 | 4.99E-03 |
| intracellular organelle part (GO:0044446) | 132 | 1.48E-10 |
| organelle part (GO:0044422) | 132 | 1.71E-10 |
| organelle membrane (GO:0031090) | 54 | 5.02E-03 |
| cytosol (GO:0005829) | 58 | 3.78E-03 |
| chloroplast (GO:0009507) | 120 | 8.20E-07 |
| plastid (GO:0009536) | 120 | 2.02E-06 |
| cytoplasmic part (GO:0044444) | 247 | 4.71E-05 |
| cytoplasm (GO:0005737) | 292 | 2.37E-04 |
|  |  |  |

GO-terms of C genome dominance genes shared among hybrids and allopolyploid

| GO | Gene count | P value |
| --- | --- | --- |
| GO molecular function complete |  |  |
| structural constituent of ribosome (GO:0003735) | 25 | 5.54E-03 |
| protein binding (GO:0005515) | 94 | 3.30E-02 |
| heterocyclic compound binding (GO:1901363) | 179 | 4.05E-02 |
| binding (GO:0005488) | 287 | 2.81E-02 |
| Unclassified | 237 | 0.00E+00 |
|  |  |  |
| GO biological process complete |  |  |
| oxidoreduction coenzyme metabolic process (GO:0006733) | 27 | 1.82E-03 |
| pyridine-containing compound metabolic process (GO:0072524) | 23 | 2.27E-02 |
| pyruvate metabolic process (GO:0006090) | 24 | 2.37E-02 |
| response to cadmium ion (GO:0046686) | 26 | 1.05E-02 |
| coenzyme metabolic process (GO:0006732) | 35 | 6.31E-04 |
| cellular aldehyde metabolic process (GO:0006081) | 24 | 4.29E-02 |
| cofactor metabolic process (GO:0051186) | 42 | 7.82E-05 |
| response to salt stress (GO:0009651) | 36 | 2.35E-03 |
| response to metal ion (GO:0010038) | 30 | 2.50E-02 |
| response to osmotic stress (GO:0006970) | 37 | 4.22E-03 |
| organophosphate metabolic process (GO:0019637) | 52 | 3.01E-02 |
| single-organism carbohydrate metabolic process (GO:0044723) | 56 | 2.68E-02 |
| organonitrogen compound metabolic process (GO:1901564) | 93 | 3.59E-04 |
| oxoacid metabolic process (GO:0043436) | 79 | 1.83E-02 |
| organic acid metabolic process (GO:0006082) | 79 | 1.91E-02 |
| phosphate-containing compound metabolic process (GO:0006796) | 85 | 2.05E-02 |
| small molecule metabolic process (GO:0044281) | 104 | 3.00E-03 |
| phosphorus metabolic process (GO:0006793) | 86 | 3.90E-02 |
| response to chemical (GO:0042221) | 127 | 2.07E-03 |
| cellular protein metabolic process (GO:0044267) | 132 | 3.46E-03 |
| cellular component organization or biogenesis (GO:0071840) | 140 | 4.30E-03 |
| protein metabolic process (GO:0019538) | 145 | 2.88E-03 |
| cellular nitrogen compound metabolic process (GO:0034641) | 165 | 8.07E-04 |
| heterocycle metabolic process (GO:0046483) | 152 | 3.41E-03 |
| cellular component organization (GO:0016043) | 125 | 3.99E-02 |
| nitrogen compound metabolic process (GO:0006807) | 177 | 9.76E-04 |
| cellular macromolecule metabolic process (GO:0044260) | 223 | 7.38E-05 |
| macromolecule metabolic process (GO:0043170) | 242 | 2.42E-05 |
| biosynthetic process (GO:0009058) | 194 | 3.10E-03 |
| cellular biosynthetic process (GO:0044249) | 179 | 1.09E-02 |
| primary metabolic process (GO:0044238) | 309 | 4.33E-07 |
| cellular metabolic process (GO:0044237) | 307 | 1.02E-06 |
| organic substance biosynthetic process (GO:1901576) | 183 | 1.54E-02 |
| organic substance metabolic process (GO:0071704) | 330 | 2.45E-07 |
| response to stimulus (GO:0050896) | 204 | 7.64E-03 |
| cellular process (GO:0009987) | 383 | 2.45E-06 |
| metabolic process (GO:0008152) | 351 | 1.63E-04 |
| Unclassified | 227 | 0.00E+00 |
|  |  |  |
| GO cellular component complete |  |  |
| chloroplast membrane (GO:0031969) | 14 | 4.73E-02 |
| plastid envelope (GO:0009526) | 36 | 1.61E-05 |
| chloroplast envelope (GO:0009941) | 35 | 2.71E-05 |
| cytosolic part (GO:0044445) | 23 | 4.80E-03 |
| ribosomal subunit (GO:0044391) | 20 | 1.83E-02 |
| cytosolic ribosome (GO:0022626) | 21 | 1.37E-02 |
| chloroplast stroma (GO:0009570) | 35 | 8.27E-05 |
| plastid stroma (GO:0009532) | 36 | 6.12E-05 |
| ribosome (GO:0005840) | 29 | 1.38E-03 |
| envelope (GO:0031975) | 56 | 8.32E-08 |
| organelle envelope (GO:0031967) | 55 | 1.88E-07 |
| chloroplast part (GO:0044434) | 65 | 9.77E-09 |
| plastid part (GO:0044435) | 65 | 2.09E-08 |
| ribonucleoprotein complex (GO:0030529) | 33 | 1.29E-02 |
| cytosol (GO:0005829) | 84 | 3.48E-07 |
| organelle part (GO:0044422) | 168 | 8.17E-13 |
| intracellular organelle part (GO:0044446) | 167 | 1.44E-12 |
| organelle membrane (GO:0031090) | 67 | 2.26E-03 |
| macromolecular complex (GO:0032991) | 83 | 1.83E-03 |
| chloroplast (GO:0009507) | 149 | 2.65E-07 |
| plastid (GO:0009536) | 150 | 4.20E-07 |
| membrane (GO:0016020) | 218 | 2.75E-06 |
| cytoplasmic part (GO:0044444) | 303 | 7.97E-04 |
| cytoplasm (GO:0005737) | 354 | 3.22E-02 |
| organelle (GO:0043226) | 478 | 2.93E-02 |
| intracellular organelle (GO:0043229) | 477 | 3.87E-02 |
| Unclassified | 200 | 0.00E+00 |

GO-slims of shared dominance genes between AC1 and AC2 (pattern XII)

| GO-slim | Gene count | P value |
| --- | --- | --- |
| Molecular Function |  |  |
| structural constituent of ribosome | 40 | 2.05E-09 |
| isomerase activity | 24 | 1.95E-02 |
| structural molecule activity | 57 | 5.59E-04 |
|  |  |  |
| Biological Process |  |  |
| translation | 62 | 6.17E-07 |
| protein metabolic process | 179 | 3.45E-04 |
| metabolic process | 388 | 2.00E-05 |
| primary metabolic process | 316 | 1.03E-03 |
|  |  |  |
| Cellular Component |  |  |
| cytoplasm | 52 | 1.66E-03 |

GO-terms of shared dominance genes between AC1 and AC2 (pattern XIII)

| GO-terms | Gene count | P value |
| --- | --- | --- |
| GO biological process complete |  |  |
| response to water deprivation | 13 | 4.86E-03 |
| response to water | 13 | 5.98E-03 |
| regulation of innate immune response | 14 | 6.32E-03 |
| regulation of immune response | 14 | 6.55E-03 |
| regulation of immune system process | 14 | 7.31E-03 |
| response to alcohol | 20 | 1.44E-04 |
| regulation of programmed cell death | 13 | 2.95E-02 |
| regulation of cell death | 13 | 3.61E-02 |
| response to abscisic acid | 16 | 6.52E-03 |
| response to lipid | 21 | 4.83E-04 |
| regulation of defense response | 15 | 4.37E-02 |
| response to osmotic stress | 17 | 4.38E-02 |
| response to nitrogen compound | 19 | 1.93E-02 |
| response to acid chemical | 34 | 8.79E-06 |
| response to oxygen-containing compound | 48 | 4.33E-08 |
| response to inorganic substance | 27 | 2.35E-03 |
| response to hormone | 32 | 1.44E-03 |
| response to endogenous stimulus | 35 | 9.90E-04 |
| response to chemical | 63 | 4.68E-07 |
| response to organic substance | 46 | 2.94E-04 |
| response to abiotic stimulus | 40 | 1.18E-02 |
| response to stress | 61 | 1.32E-04 |
| response to stimulus | 92 | 1.71E-06 |

GO-slims of shared dominance genes between AC1 and AC2 (pattern XVIII)

| GO-slim | Gene count | | P value |
| --- | --- | --- | --- |
| Molecular Function | |  |  |
| structural constituent of ribosome | | 51 | 3.96E-09 |
| structural molecule activity | | 78 | 1.00E-03 |
|  | |  |  |
| Biological Process | |  |  |
| translation | | 83 | 1.32E-06 |
| primary metabolic process | | 483 | 1.85E-05 |
| metabolic process | | 581 | 3.57E-06 |

GO-terms of shared dominance genes between AC1 and AC2 (pattern XIX)

| GO-term | Gene count | P value |
| --- | --- | --- |
| GO biological process complete |  |  |
| response to stimulus | 141 | 2.47E-12 |
| response to stress | 105 | 7.83E-14 |
| response to chemical | 91 | 2.35E-10 |
| cellular response to stimulus | 68 | 8.13E-05 |
| response to organic substance | 75 | 3.22E-10 |
| response to abiotic stimulus | 60 | 3.99E-05 |
| cell communication | 53 | 5.56E-03 |
| response to external stimulus | 66 | 8.80E-10 |
| response to oxygen-containing compound | 68 | 2.96E-11 |
| multi-organism process | 63 | 6.65E-09 |
| signaling | 49 | 2.75E-03 |
| single organism signaling | 49 | 2.70E-03 |
| organic acid metabolic process | 46 | 1.29E-02 |
| oxoacid metabolic process | 46 | 1.25E-02 |
| signal transduction | 48 | 2.05E-03 |
| carboxylic acid metabolic process | 46 | 3.33E-03 |
| response to endogenous stimulus | 54 | 2.03E-07 |
| defense response | 61 | 7.36E-12 |
| response to biotic stimulus | 59 | 5.28E-12 |
| response to hormone | 45 | 4.27E-05 |
| response to other organism | 59 | 1.36E-12 |
| response to external biotic stimulus | 59 | 1.36E-12 |
| cellular localization | 38 | 1.13E-02 |
| cellular response to chemical stimulus | 42 | 8.71E-05 |
| establishment of localization in cell | 36 | 1.81E-02 |
| response to acid chemical | 50 | 2.97E-09 |
| small molecule biosynthetic process | 36 | 5.85E-03 |
| cellular response to organic substance | 40 | 6.41E-05 |
| intracellular transport | 36 | 2.86E-03 |
| monocarboxylic acid metabolic process | 42 | 3.99E-06 |
| response to inorganic substance | 36 | 5.67E-04 |
| single-organism intracellular transport | 36 | 3.34E-04 |
| cellular response to stress | 38 | 1.92E-05 |
| defense response to other organism | 49 | 6.69E-12 |
| organic acid biosynthetic process | 35 | 6.50E-05 |
| carboxylic acid biosynthetic process | 35 | 6.50E-05 |
| intracellular protein transport | 28 | 3.00E-02 |
| cellular response to endogenous stimulus | 28 | 1.18E-02 |
| cellular response to hormone stimulus | 28 | 1.07E-02 |
| hormone-mediated signaling pathway | 26 | 4.36E-02 |
| single-organism cellular localization | 28 | 3.02E-03 |
| protein targeting | 28 | 2.22E-03 |
| cellular response to oxygen-containing compound | 32 | 6.24E-06 |
| immune system process | 50 | 3.00E-18 |
| response to temperature stimulus | 28 | 4.93E-04 |
| response to organic cyclic compound | 32 | 2.34E-06 |
| regulation of response to stimulus | 35 | 2.07E-08 |
| response to nitrogen compound | 30 | 1.46E-05 |
| intracellular signal transduction | 23 | 3.36E-02 |
| response to lipid | 24 | 6.93E-03 |
| membrane organization | 27 | 1.67E-04 |
| organic hydroxy compound metabolic process | 25 | 1.88E-03 |
| immune response | 41 | 8.56E-14 |
| innate immune response | 41 | 6.28E-14 |
| response to fungus | 27 | 7.45E-05 |
| cellular response to acid chemical | 31 | 1.48E-07 |
| monocarboxylic acid biosynthetic process | 32 | 2.86E-09 |
| response to alcohol | 24 | 3.78E-04 |
| regulation of response to stress | 33 | 1.34E-10 |
| organic hydroxy compound biosynthetic process | 24 | 5.32E-05 |
| single-organism membrane organization | 22 | 8.60E-04 |
| regulation of defense response | 32 | 1.16E-10 |
| response to bacterium | 24 | 2.71E-05 |
| defense response, incompatible interaction | 36 | 3.94E-14 |
| response to abscisic acid | 23 | 8.87E-05 |
| cellular response to organic cyclic compound | 23 | 7.09E-05 |
| defense response to fungus | 21 | 1.19E-03 |
| response to salicylic acid | 24 | 1.14E-06 |
| systemic acquired resistance | 32 | 4.09E-13 |
| response to jasmonic acid | 22 | 1.19E-05 |
| response to organonitrogen compound | 24 | 3.29E-07 |
| regulation of cell death | 23 | 9.75E-07 |
| regulation of immune system process | 28 | 1.15E-10 |
| regulation of immune response | 27 | 6.00E-10 |
| response to chitin | 24 | 1.28E-07 |
| regulation of innate immune response | 27 | 5.57E-10 |
| regulation of programmed cell death | 23 | 6.75E-07 |
| establishment of protein localization to membrane | 22 | 1.29E-06 |
| protein localization to membrane | 22 | 1.29E-06 |
| regulation of cellular response to stress | 22 | 8.83E-07 |
| protein targeting to membrane | 22 | 7.26E-07 |
| regulation of plant-type hypersensitive response | 21 | 2.17E-06 |
| negative regulation of response to stimulus | 21 | 2.17E-06 |
| defense response to bacterium | 23 | 3.13E-08 |
| cellular response to salicylic acid stimulus | 21 | 9.78E-07 |
| salicylic acid mediated signaling pathway | 21 | 7.96E-07 |
| response to wounding | 17 | 4.07E-04 |
| response to endoplasmic reticulum stress | 23 | 7.65E-09 |
| negative regulation of defense response | 20 | 1.05E-07 |
| immune effector process | 17 | 2.49E-05 |
| benzene-containing compound metabolic process | 24 | 1.40E-11 |
| cellular response to jasmonic acid stimulus | 18 | 2.64E-06 |
| jasmonic acid mediated signaling pathway | 18 | 2.44E-06 |
| systemic acquired resistance, salicylic acid mediated signaling pathway | 17 | 5.87E-06 |
| phenol-containing compound metabolic process | 23 | 6.70E-12 |
| signal transduction by protein phosphorylation | 17 | 1.38E-06 |
| phenol-containing compound biosynthetic process | 23 | 2.18E-12 |
| salicylic acid metabolic process | 22 | 2.22E-11 |
| MAPK cascade | 17 | 9.54E-07 |
| salicylic acid biosynthetic process | 22 | 9.21E-12 |
| regulation of reactive oxygen species metabolic process | 14 | 1.03E-04 |
| regulation of hydrogen peroxide metabolic process | 14 | 2.73E-05 |
| negative regulation of cell death | 14 | 2.48E-05 |
| cellular response to topologically incorrect protein | 11 | 8.17E-03 |
| response to unfolded protein | 11 | 8.17E-03 |
| cellular response to unfolded protein | 11 | 8.17E-03 |
| endoplasmic reticulum unfolded protein response | 11 | 7.05E-03 |
| negative regulation of programmed cell death | 14 | 1.68E-05 |
| detection of stimulus | 12 | 8.21E-04 |
| regulation of multi-organism process | 13 | 2.00E-05 |
| regulation of flavonoid biosynthetic process | 10 | 5.04E-03 |
| positive regulation of response to stimulus | 10 | 2.39E-03 |
| respiratory burst | 11 | 1.78E-04 |
| respiratory burst involved in defense response | 11 | 1.78E-04 |
| positive regulation of flavonoid biosynthetic process | 9 | 8.90E-03 |
| detection of biotic stimulus | 10 | 2.85E-04 |
| amino acid import | 8 | 6.58E-03 |
| positive regulation of defense response | 7 | 4.41E-03 |
| positive regulation of immune response | 7 | 1.80E-03 |
| positive regulation of immune system process | 7 | 1.80E-03 |
| positive regulation of innate immune response | 7 | 1.21E-03 |
| activation of immune response | 7 | 3.04E-04 |
| activation of innate immune response | 7 | 3.04E-04 |
|  |  |  |
| GO molecular function complete |  |  |
| calcium ion binding | 12 | 8.90E-03 |
|  |  |  |
| GO cellular component complete |  |  |
| plasma membrane | 63 | 2.90E-02 |
| mitochondrion | 19 | 4.91E-03 |
