# Supplementary material for: Genome-Wide Gene Expressions Respond Differently to A-subgenome Origins in Brassica napus Synthetic Hybrids and Natural Allotetraploid
Source: Front Plant Sci. 2016 Oct 13;7:1508. doi: 10.3389/fpls.2016.01508 (PMC5061818; doi:10.3389/fpls.2016.01508)
Supplement: Table S5 — GO analysis of genes showing homoeolog expression bias in two hybrids. [file Table5.DOC]

**Table S5 GO analysis of genes showing homoeolog expression bias in two hybrids.**

GO-slims of shared A-bias genes in both AC1 and AC2

| GO-Slim | Gene count | P value |
| --- | --- | --- |
| Molecular Function |  |  |
| structural molecule activity | 37 | 2.64E-03 |
| kinase activity | 11 | 3.90E-02 |
| protein kinase activity | 4 | 1.87E-03 |
| transmembrane receptor protein kinase activity | 1 | 1.27E-02 |
|  |  |  |
| Biological Process |  |  |
| generation of precursor metabolites and energy | 22 | 3.57E-02 |
| cellular protein modification process | 17 | 2.13E-02 |
| protein phosphorylation | 7 | 8.18E-03 |
|  |  |  |
| Cellular Component |  |  |
| cytosol | 7 | 8.33E-03 |
| ribosome | 9 | 1.47E-03 |

GO-slims of shared C-bias genes in both AC1 and AC2

| GO-Slim | Gene count | P value |
| --- | --- | --- |
| Molecular Function |  |  |
| structural constituent of ribosome | 27 | 1.20E-07 |
| structural molecule activity | 49 | 1.21E-08 |
| oxidoreductase activity | 51 | 3.12E-04 |
| kinase activity | 9 | 6.66E-03 |
| protein kinase activity | 6 | 3.75E-02 |
|  |  |  |
| Biological Process |  |  |
| mitochondrial transport | 6 | 3.65E-02 |
| mitochondrion organization | 7 | 1.87E-02 |
| translation | 35 | 1.73E-03 |
| cellular protein modification process | 12 | 2.34E-04 |
| protein phosphorylation | 6 | 3.00E-03 |
|  |  |  |
| Cellular Component |  |  |
| ribosome | 11 | 2.67E-05 |
| cytosol | 8 | 1.06E-03 |
| ribonucleoprotein complex | 14 | 1.65E-02 |
| cytoplasm | 32 | 1.33E-02 |

GO-terms of A-bias genes showing only in AC1

| GO-Term | Gene count | P value |
| --- | --- | --- |
| GO biological process |  |  |
| root hair elongation | 11 | 3.37E-02 |
| photosynthesis, light reaction | 14 | 1.00E-02 |
| photosynthesis | 16 | 2.84E-02 |
| generation of precursor metabolites and energy | 21 | 2.10E-02 |
| monocarboxylic acid biosynthetic process | 22 | 4.39E-02 |
| organic acid biosynthetic process | 33 | 1.19E-02 |
| carboxylic acid biosynthetic process | 33 | 1.19E-02 |
| small molecule biosynthetic process | 41 | 1.46E-03 |
| response to acid chemical | 42 | 1.49E-03 |
| monocarboxylic acid metabolic process | 36 | 4.84E-02 |
| organonitrogen compound biosynthetic process | 45 | 4.66E-03 |
| response to hormone | 43 | 1.09E-02 |
| response to endogenous stimulus | 48 | 4.79E-03 |
| response to oxygen-containing compound | 57 | 5.53E-04 |
| response to organic substance | 70 | 2.45E-05 |
| organonitrogen compound metabolic process | 60 | 4.73E-04 |
| carboxylic acid metabolic process | 48 | 2.44E-02 |
| response to chemical | 89 | 3.32E-06 |
| response to abiotic stimulus | 57 | 3.62E-02 |
| response to stimulus | 121 | 3.99E-02 |
| cellular process | 213 | 1.38E-02 |
|  |  |  |
| GO cellular component |  |  |
| cytosolic small ribosomal subunit | 8 | 4.08E-02 |
| chloroplast thylakoid | 20 | 8.27E-05 |
| plastid thylakoid | 20 | 8.27E-05 |
| chloroplast thylakoid membrane | 14 | 1.19E-02 |
| plastid thylakoid membrane | 14 | 1.25E-02 |
| thylakoid | 21 | 1.72E-04 |
| thylakoid part | 16 | 6.00E-03 |
| cytosolic ribosome | 15 | 1.17E-02 |
| plant-type cell wall | 14 | 2.30E-02 |
| thylakoid membrane | 14 | 2.30E-02 |
| photosynthetic membrane | 14 | 2.41E-02 |
| cytosolic part | 15 | 2.78E-02 |
| apoplast | 18 | 6.71E-03 |
| organelle subcompartment | 27 | 9.68E-05 |
| chloroplast envelope | 21 | 3.57E-03 |
| plastid envelope | 21 | 5.10E-03 |
| external encapsulating structure | 25 | 2.45E-03 |
| cell wall | 24 | 6.39E-03 |
| chloroplast part | 35 | 7.54E-04 |
| plastid part | 35 | 1.13E-03 |
| envelope | 28 | 1.73E-02 |
| organelle envelope | 27 | 3.90E-02 |
| intracellular organelle part | 77 | 4.38E-02 |
| organelle part | 77 | 4.67E-02 |

GO-terms of A-bias genes showing only in AC2

| GO-Term | Gene count | P value |
| --- | --- | --- |
| GO molecular function |  |  |
| structural constituent of ribosome | 33 | 2.09E-11 |
| poly(A) RNA binding | 15 | 5.67E-04 |
| structural molecule activity | 35 | 6.32E-10 |
|  |  |  |
| GO biological process |  |  |
| cytoplasmic translation | 8 | 1.74E-03 |
| electron transport chain | 9 | 2.05E-02 |
| ribosome biogenesis | 31 | 1.48E-09 |
| isopentenyl diphosphate biosynthetic process, methylerythritol 4-phosphate pathway | 16 | 6.30E-04 |
| isopentenyl diphosphate metabolic process | 16 | 9.53E-04 |
| isopentenyl diphosphate biosynthetic process | 16 | 9.53E-04 |
| chloroplast organization | 14 | 6.12E-03 |
| ribonucleoprotein complex biogenesis | 31 | 1.67E-08 |
| RNA methylation | 13 | 3.16E-02 |
| rRNA processing | 16 | 3.75E-03 |
| rRNA metabolic process | 16 | 4.32E-03 |
| plastid organization | 21 | 1.80E-04 |
| pyruvate metabolic process | 25 | 1.18E-05 |
| glyceraldehyde-3-phosphate metabolic process | 19 | 1.23E-03 |
| RNA modification | 23 | 9.18E-05 |
| translation | 38 | 2.28E-08 |
| peptide biosynthetic process | 38 | 3.05E-08 |
| ncRNA processing | 18 | 1.14E-02 |
| amide biosynthetic process | 39 | 5.45E-08 |
| peptide metabolic process | 38 | 1.19E-07 |
| ncRNA metabolic process | 22 | 3.27E-03 |
| cellular amide metabolic process | 40 | 2.30E-07 |
| nicotinamide nucleotide metabolic process | 18 | 3.80E-02 |
| photosynthesis | 18 | 3.80E-02 |
| pyridine nucleotide metabolic process | 18 | 4.19E-02 |
| generation of precursor metabolites and energy | 28 | 2.51E-04 |
| pyridine-containing compound metabolic process | 18 | 4.84E-02 |
| cellular aldehyde metabolic process | 20 | 1.86E-02 |
| oxidoreduction coenzyme metabolic process | 20 | 2.04E-02 |
| glycosyl compound metabolic process | 23 | 2.19E-02 |
| coenzyme metabolic process | 25 | 2.09E-02 |
| ribose phosphate metabolic process | 24 | 3.82E-02 |
| cofactor metabolic process | 30 | 4.38E-03 |
| organophosphate biosynthetic process | 35 | 9.34E-04 |
| organonitrogen compound biosynthetic process | 65 | 1.30E-07 |
| organophosphate metabolic process | 47 | 8.04E-05 |
| nucleotide metabolic process | 29 | 4.23E-02 |
| nucleoside phosphate metabolic process | 29 | 4.37E-02 |
| organonitrogen compound metabolic process | 89 | 9.12E-11 |
| nucleobase-containing small molecule metabolic process | 30 | 3.83E-02 |
| carbohydrate derivative metabolic process | 50 | 1.54E-04 |
| monocarboxylic acid metabolic process | 47 | 1.14E-03 |
| cellular component biogenesis | 52 | 2.89E-04 |
| carboxylic acid metabolic process | 61 | 1.31E-03 |
| oxoacid metabolic process | 65 | 6.17E-04 |
| organic acid metabolic process | 65 | 6.45E-04 |
| small molecule metabolic process | 86 | 2.27E-05 |
| response to abiotic stimulus | 74 | 7.11E-04 |
| single-organism biosynthetic process | 85 | 4.95E-03 |
| biosynthetic process | 147 | 6.38E-04 |
| cellular nitrogen compound metabolic process | 117 | 2.20E-02 |
| organic substance biosynthetic process | 139 | 2.78E-03 |
| cellular biosynthetic process | 134 | 5.78E-03 |
| nitrogen compound metabolic process | 127 | 1.22E-02 |
|  |  |  |
| GO cellular component |  |  |
| cytosolic large ribosomal subunit | 16 | 1.62E-06 |
| large ribosomal subunit | 18 | 1.02E-06 |
| respiratory chain | 9 | 2.97E-02 |
| cytosolic ribosome | 27 | 4.67E-09 |
| ribosomal subunit | 25 | 3.98E-08 |
| ribosome | 37 | 4.64E-12 |
| inner mitochondrial membrane protein complex | 10 | 2.63E-02 |
| cytosolic part | 27 | 2.57E-08 |
| mitochondrial protein complex | 10 | 3.36E-02 |
| thylakoid | 28 | 6.52E-07 |
| thylakoid part | 21 | 1.55E-04 |
| ribonucleoprotein complex | 39 | 2.61E-09 |
| thylakoid membrane | 18 | 1.62E-03 |
| photosynthetic membrane | 18 | 1.72E-03 |
| chloroplast envelope | 30 | 3.09E-06 |
| chloroplast thylakoid membrane | 16 | 1.06E-02 |
| plastid thylakoid membrane | 16 | 1.12E-02 |
| chloroplast thylakoid | 21 | 7.52E-04 |
| plastid thylakoid | 21 | 7.52E-04 |
| plastid envelope | 30 | 5.26E-06 |
| nucleolus | 15 | 2.68E-02 |
| organelle inner membrane | 17 | 9.32E-03 |
| organelle envelope | 44 | 1.93E-07 |
| envelope | 44 | 2.29E-07 |
| plastid part | 52 | 1.73E-08 |
| chloroplast part | 51 | 2.77E-08 |
| vacuole | 40 | 6.08E-06 |
| plastid stroma | 25 | 5.32E-03 |
| membrane protein complex | 22 | 2.31E-02 |
| chloroplast stroma | 23 | 2.50E-02 |
| organelle subcompartment | 26 | 1.78E-02 |
| intracellular non-membrane-bounded organelle | 45 | 9.22E-05 |
| non-membrane-bounded organelle | 45 | 9.22E-05 |
| macromolecular complex | 75 | 2.27E-07 |
| plastid | 137 | 3.53E-15 |
| cytosol | 61 | 1.94E-05 |
| chloroplast | 133 | 3.81E-14 |
| intracellular organelle part | 127 | 6.96E-12 |
| organelle part | 127 | 8.05E-12 |
| cytoplasmic part | 239 | 1.69E-07 |
| cytoplasm | 267 | 5.09E-04 |

GO-terms of C-bias genes showing only in AC1

| GO-Term | Gene count | P value |
| --- | --- | --- |
| GO molecular function |  |  |
| structural constituent of ribosome | 28 | 3.08E-08 |
|  |  |  |
| GO biological process |  |  |
| response to red light | 9 | 1.43E-02 |
| ribosome biogenesis | 31 | 4.58E-10 |
| cysteine biosynthetic process | 14 | 3.37E-03 |
| glucose 6-phosphate metabolic process | 12 | 1.84E-02 |
| pentose-phosphate shunt | 12 | 1.84E-02 |
| cysteine metabolic process | 14 | 4.29E-03 |
| rRNA processing | 17 | 4.29E-04 |
| NADP metabolic process | 12 | 2.53E-02 |
| serine family amino acid biosynthetic process | 14 | 5.01E-03 |
| ribonucleoprotein complex biogenesis | 31 | 5.32E-09 |
| rRNA metabolic process | 17 | 5.00E-04 |
| plastid organization | 22 | 1.81E-05 |
| glyceraldehyde-3-phosphate metabolic process | 20 | 1.39E-04 |
| photosynthesis | 22 | 8.21E-05 |
| serine family amino acid metabolic process | 15 | 1.18E-02 |
| response to cytokinin | 15 | 1.44E-02 |
| photosynthesis, light reaction | 15 | 1.85E-02 |
| cellular aldehyde metabolic process | 22 | 7.07E-04 |
| ncRNA processing | 17 | 2.37E-02 |
| ncRNA metabolic process | 21 | 5.66E-03 |
| translation | 32 | 1.89E-05 |
| peptide biosynthetic process | 32 | 2.40E-05 |
| amide biosynthetic process | 34 | 9.85E-06 |
| peptide metabolic process | 32 | 7.12E-05 |
| glucan metabolic process | 19 | 4.34E-02 |
| cellular glucan metabolic process | 19 | 4.34E-02 |
| cellular amide metabolic process | 35 | 2.97E-05 |
| sulfur compound biosynthetic process | 21 | 3.21E-02 |
| response to osmotic stress | 28 | 7.74E-03 |
| response to salt stress | 26 | 1.88E-02 |
| organonitrogen compound biosynthetic process | 60 | 2.59E-06 |
| cellular component biogenesis | 53 | 2.75E-05 |
| organonitrogen compound metabolic process | 74 | 6.99E-06 |
| response to organic substance | 77 | 2.21E-04 |
| response to abiotic stimulus | 66 | 2.87E-02 |
| cellular nitrogen compound biosynthetic process | 80 | 4.39E-03 |
| cellular component organization or biogenesis | 108 | 7.11E-05 |
| single-organism transport | 79 | 2.45E-02 |
| single-organism localization | 79 | 3.62E-02 |
| response to chemical | 89 | 1.43E-02 |
| biosynthetic process | 143 | 3.05E-04 |
| cellular biosynthetic process | 131 | 2.16E-03 |
| organic substance biosynthetic process | 133 | 4.34E-03 |
| cellular process | 257 | 1.18E-03 |
|  |  |  |
| GO cellular component |  |  |
| plastoglobule | 7 | 1.02E-02 |
| cytosolic large ribosomal subunit | 14 | 4.33E-05 |
| large ribosomal subunit | 15 | 1.20E-04 |
| ribosomal subunit | 23 | 4.50E-07 |
| cytosolic ribosome | 24 | 2.65E-07 |
| cytosolic part | 24 | 1.17E-06 |
| ribosome | 29 | 3.29E-07 |
| chloroplast thylakoid | 22 | 9.54E-05 |
| plastid thylakoid | 22 | 9.54E-05 |
| plastid stroma | 33 | 1.22E-07 |
| chloroplast stroma | 30 | 3.23E-06 |
| thylakoid | 23 | 2.51E-04 |
| organelle inner membrane | 16 | 1.91E-02 |
| thylakoid part | 17 | 1.56E-02 |
| ribonucleoprotein complex | 32 | 7.34E-06 |
| thylakoid membrane | 15 | 4.55E-02 |
| photosynthetic membrane | 15 | 4.77E-02 |
| plastid part | 54 | 3.08E-10 |
| chloroplast envelope | 25 | 6.31E-04 |
| plastid envelope | 25 | 9.61E-04 |
| chloroplast part | 51 | 5.47E-09 |
| envelope | 38 | 4.53E-05 |
| membrane protein complex | 21 | 3.42E-02 |
| organelle envelope | 37 | 1.10E-04 |
| organelle subcompartment | 26 | 8.42E-03 |
| intracellular non-membrane-bounded organelle | 42 | 4.20E-04 |
| non-membrane-bounded organelle | 42 | 4.20E-04 |
| macromolecular complex | 71 | 9.62E-07 |
| intracellular organelle part | 122 | 1.54E-11 |
| organelle part | 122 | 1.77E-11 |
| cytosol | 51 | 9.80E-03 |
| chloroplast | 98 | 4.92E-04 |
| plastid | 99 | 5.46E-04 |
| membrane | 142 | 5.47E-03 |
| cytoplasmic part | 203 | 3.94E-02 |

GO-terms of C-bias genes showing only in AC2

| GO-Term | Gene count | P value |
| --- | --- | --- |
| GO molecular function |  |  |
| amide binding | 7 | 4.33E-02 |
| metal cluster binding | 10 | 1.63E-02 |
| iron-sulfur cluster binding | 10 | 1.63E-02 |
| structural constituent of ribosome | 27 | 3.27E-07 |
| structural molecule activity | 29 | 3.39E-06 |
| phosphotransferase activity, alcohol group as acceptor | 1 | 9.88E-03 |
|  |  |  |
|  |  |  |
| GO biological process |  |  |
| glycine catabolic process | 7 | 3.15E-02 |
| serine family amino acid catabolic process | 7 | 4.39E-02 |
| chlorophyll biosynthetic process | 11 | 1.99E-03 |
| cysteine metabolic process | 20 | 1.41E-07 |
| cysteine biosynthetic process | 19 | 6.89E-07 |
| serine family amino acid biosynthetic process | 19 | 1.21E-06 |
| porphyrin-containing compound biosynthetic process | 12 | 2.57E-03 |
| tetrapyrrole biosynthetic process | 12 | 3.17E-03 |
| serine family amino acid metabolic process | 22 | 1.76E-07 |
| chlorophyll metabolic process | 14 | 7.07E-04 |
| isopentenyl diphosphate biosynthetic process, methylerythritol 4-phosphate pathway | 18 | 1.75E-05 |
| isopentenyl diphosphate metabolic process | 18 | 2.82E-05 |
| isopentenyl diphosphate biosynthetic process | 18 | 2.82E-05 |
| glucose 6-phosphate metabolic process | 13 | 4.89E-03 |
| pentose-phosphate shunt | 13 | 4.89E-03 |
| glyceraldehyde-3-phosphate metabolic process | 24 | 4.06E-07 |
| porphyrin-containing compound metabolic process | 15 | 1.04E-03 |
| tetrapyrrole metabolic process | 15 | 1.14E-03 |
| NADP metabolic process | 13 | 6.92E-03 |
| response to cytokinin | 19 | 4.35E-05 |
| maltose metabolic process | 11 | 3.89E-02 |
| sulfur amino acid biosynthetic process | 23 | 1.81E-06 |
| sulfur amino acid metabolic process | 25 | 4.18E-07 |
| leaf morphogenesis | 14 | 8.01E-03 |
| cellular aldehyde metabolic process | 28 | 2.17E-07 |
| starch biosynthetic process | 13 | 2.11E-02 |
| RNA methylation | 13 | 2.83E-02 |
| disaccharide metabolic process | 13 | 3.76E-02 |
| pyruvate metabolic process | 25 | 9.55E-06 |
| alpha-amino acid biosynthetic process | 27 | 2.66E-06 |
| oligosaccharide metabolic process | 14 | 2.87E-02 |
| starch metabolic process | 14 | 3.27E-02 |
| sulfur compound biosynthetic process | 29 | 1.94E-06 |
| response to cadmium ion | 25 | 4.45E-05 |
| cellular amino acid biosynthetic process | 29 | 3.60E-06 |
| nicotinamide nucleotide metabolic process | 21 | 6.17E-04 |
| pyridine nucleotide metabolic process | 21 | 6.97E-04 |
| cofactor biosynthetic process | 16 | 1.79E-02 |
| pyridine-containing compound metabolic process | 21 | 8.33E-04 |
| shoot system morphogenesis | 26 | 4.90E-05 |
| oxidoreduction coenzyme metabolic process | 23 | 3.67E-04 |
| response to metal ion | 31 | 3.58E-06 |
| glucan biosynthetic process | 16 | 3.19E-02 |
| pigment metabolic process | 16 | 4.68E-02 |
| ribosome biogenesis | 21 | 3.53E-03 |
| plastid organization | 17 | 3.86E-02 |
| ribonucleoprotein complex biogenesis | 22 | 4.52E-03 |
| phospholipid biosynthetic process | 21 | 7.90E-03 |
| alpha-amino acid metabolic process | 30 | 1.07E-04 |
| sulfur compound metabolic process | 32 | 4.42E-05 |
| ribose phosphate metabolic process | 28 | 3.42E-04 |
| cofactor metabolic process | 34 | 4.13E-05 |
| phospholipid metabolic process | 22 | 1.71E-02 |
| peptide metabolic process | 31 | 4.88E-04 |
| translation | 29 | 1.40E-03 |
| peptide biosynthetic process | 29 | 1.71E-03 |
| cellular amino acid metabolic process | 33 | 8.54E-04 |
| organonitrogen compound biosynthetic process | 71 | 1.60E-10 |
| nucleotide metabolic process | 32 | 1.76E-03 |
| nucleoside phosphate metabolic process | 32 | 1.82E-03 |
| amide biosynthetic process | 29 | 6.26E-03 |
| organophosphate biosynthetic process | 35 | 7.31E-04 |
| coenzyme metabolic process | 24 | 5.00E-02 |
| cellular amide metabolic process | 31 | 5.09E-03 |
| response to inorganic substance | 51 | 2.97E-06 |
| nucleobase-containing small molecule metabolic process | 32 | 4.54E-03 |
| organophosphate metabolic process | 48 | 2.19E-05 |
| organic acid biosynthetic process | 42 | 3.97E-04 |
| carboxylic acid biosynthetic process | 42 | 3.97E-04 |
| organonitrogen compound metabolic process | 89 | 4.87E-11 |
| carboxylic acid metabolic process | 71 | 1.97E-07 |
| monocarboxylic acid metabolic process | 48 | 3.50E-04 |
| oxoacid metabolic process | 73 | 5.79E-07 |
| organic acid metabolic process | 73 | 6.12E-07 |
| small molecule biosynthetic process | 46 | 5.58E-03 |
| carbohydrate derivative metabolic process | 45 | 8.25E-03 |
| small molecule metabolic process | 88 | 3.01E-06 |
| response to chemical | 108 | 3.13E-07 |
| response to external stimulus | 64 | 8.63E-03 |
| response to organic substance | 75 | 3.34E-03 |
| single-organism biosynthetic process | 86 | 1.74E-03 |
| cellular biosynthetic process | 132 | 8.61E-03 |
| response to stimulus | 151 | 3.55E-03 |
| biosynthetic process | 137 | 4.14E-02 |
| single-organism cellular process | 191 | 8.51E-03 |
| cellular process | 259 | 1.37E-02 |
|  |  |  |
| GO cellular component |  |  |
| mitochondrial large ribosomal subunit | 5 | 2.68E-03 |
| organellar large ribosomal subunit | 5 | 5.59E-03 |
| mitochondrial ribosome | 5 | 4.67E-02 |
| large ribosomal subunit | 18 | 8.68E-07 |
| mitochondrial matrix | 9 | 3.29E-02 |
| ribosomal subunit | 24 | 1.69E-07 |
| cytosolic large ribosomal subunit | 11 | 1.24E-02 |
| chloroplast stroma | 41 | 8.98E-13 |
| plastid stroma | 41 | 2.57E-12 |
| ribosome | 31 | 4.03E-08 |
| cytosolic ribosome | 19 | 8.51E-04 |
| cytosolic part | 20 | 6.84E-04 |
| ribonucleoprotein complex | 36 | 1.09E-07 |
| chloroplast part | 54 | 5.87E-10 |
| plastid envelope | 26 | 5.62E-04 |
| plastid part | 54 | 1.18E-09 |
| chloroplast envelope | 25 | 1.15E-03 |
| thylakoid | 20 | 1.58E-02 |
| organelle envelope | 35 | 1.70E-03 |
| envelope | 35 | 1.91E-03 |
| vacuole | 31 | 2.87E-02 |
| intracellular non-membrane-bounded organelle | 40 | 5.32E-03 |
| non-membrane-bounded organelle | 40 | 5.32E-03 |
| intracellular organelle part | 127 | 3.03E-12 |
| organelle part | 127 | 3.52E-12 |
| cytosol | 57 | 3.33E-04 |
| chloroplast | 124 | 3.88E-11 |
| macromolecular complex | 68 | 4.30E-05 |
| plastid | 125 | 5.10E-11 |
| cytoplasmic part | 236 | 2.68E-07 |
| cytoplasm | 279 | 5.73E-07 |
| nucleus | 119 | 1.21E-02 |
