# Supplementary material for: Genome-Wide Gene Expressions Respond Differently to A-subgenome Origins in Brassica napus Synthetic Hybrids and Natural Allotetraploid
Source: Front Plant Sci. 2016 Oct 13;7:1508. doi: 10.3389/fpls.2016.01508 (PMC5061818; doi:10.3389/fpls.2016.01508)
Supplement: Table S6 — The corresponding primers of qRT-PCR. [file Table6.DOC]

**Supplementary Table 6.The corresponding primers of qRT-PCR**

| **Gene** | **Primer sequences (Left)** | **Primer sequences (Right)** |
| --- | --- | --- |
| BnaA06g34470D | TGGCACGTACATGGAGGATT | CTGACGACGTCCGATGATGT |
| BnaAnng38970D | CAATGTGGAGGGGAATGC | GTTTGTTGCCGTAAGTGCCT |
| BnaC03g30720D | GACGGTGGAAGCGTGAGAT | GGAGATACTGGCGGAGTTGA |
| BnaA09g13300D | GGATTGATGTTCCTCCACTCA | CTGAAACTTGACCCCAGCAT |
| BnaA03g55000D | AGTGCCAAAAGTACAGCCCA | GGCCTTTACCGATCTCTACCC |
| BnaA03g46100D | TGATGAGAGACGAAACGGTG | TTTTGCAGGCTCTGGTTCTT |
| BnaC01g32940D | CAGGGGACATAGGGAAAACA | TCGTCTCAGGCAACATCAAG |
| BnaC03g73590D | CTTAACCGCAGCTACACGGA | CGGTGTCGGTGGATGTGTTA |
| BnaC03g74010D | GCCACTGCTTCAGTACGACC | ACATGTAGCGGAGTGAACCG |
| BnaA02g04960D | CTGGTGCGTCTGAGGTTCTT | CTTCCGAAGGCAACAAAGGC |
| BnaA03g11650D | GTGTTAGTGCTGTGTGTGCG | TGTCACTGGTTTATCTCCTTTGTT |
| BnaA02g10130D | TCGGTCAGCCGGTAGAGTAA | TGTTCGGTCCAAAAGCCACT |
| BnaC08g11600D | TCTACGCCCTTTTGGGTACA | CAATTGTGAGGGGACGAAGGA |
| BnaC03g30930D | TGAGGGTGGTGATGGTGACT | CTGTACCACGGTATCTCACGAT |
| BnaAnng29770D | TCGTCACGTCACCATCAGTG | TGTACGGCCATGAAACACCA |
| BnaC01g39450D | GCTGACTTGGGCGACTCATA | CCTCCGTCTTCTCCTCCTCA |
| BnaA02g07860D | ACGACCAGCTTGATACAGCG | CTGCAGCCATCACCAACGAC |
| BnaAnng24470D | TGGTTGCCCCAATTGATGTT | GGGTGATGATTTCCACTGTTCCT |
| BnaC03g72450D | TCGTGAAAACCCTAACGGGG | TGGATGTTGTAGTCGGCGAG |
| BnaC03g72540D | CCGGTTTCTCTCTCGGATCG | TCTGCGCTTCCGATACCATC |
| BnaA04g18600D | CGGAGCCAGTTGTGTCGTTTAAT | CGGTGGCGTCTTTAGAGGG |
| BnaCnng21680D | AGAGATTGGGACGCTGACGA | CTCCGCAGCCAAAAGTGAAG |
| Actin of *B. napus* | TCCATCCATCGTCCACAG | GCATCATCACAAGCATCCTT |
